# Human B Cell Differentiation Is Characterized by Progressive Remodeling of O-Linked Glycans

**DOI:** 10.3389/fimmu.2018.02857

**Published:** 2018-12-14

**Authors:** Nicholas Giovannone, Aristotelis Antonopoulos, Jennifer Liang, Jenna Geddes Sweeney, Matthew R. Kudelka, Sandra L. King, Gi Soo Lee, Richard D. Cummings, Anne Dell, Steven R. Barthel, Hans R. Widlund, Stuart M. Haslam, Charles J. Dimitroff

**Affiliations:** ^1^Department of Dermatology, Brigham and Women's Hospital, Boston MA, United States; ^2^Harvard Medical School, Boston MA, United States; ^3^Department of Life Sciences, Imperial College London, London, United Kingdom; ^4^Department of Surgery, Beth Israel Deaconess Medical Center, Boston, MA, United States; ^5^Department of Biochemistry, Emory University School of Medicine, Atlanta, GA, United States; ^6^Department of Otology and Laryngology, Harvard Medical School, Bostonm, MA, United States

**Keywords:** glycosylation, glycan, B cell, CD45, peanut lectin, PNA, ST3Gal1, GCNT1

## Abstract

Germinal centers (GC) are microanatomical niches where B cells proliferate, undergo antibody affinity maturation, and differentiate to long-lived memory B cells and antibody-secreting plasma cells. For decades, GC B cells have been defined by their reactivity to the plant lectin peanut agglutinin (PNA), which binds serine/threonine (O-linked) glycans containing the asialylated disaccharide Gal-β1,3-GalNAc-Ser/Thr (also called T-antigen). In T cells, acquisition of PNA binding by activated T cells and thymocytes has been linked with altered tissue homing patterns, cell signaling, and survival. Yet, in GC B cells, the glycobiological basis and significance of PNA binding remains surprisingly unresolved. Here, we investigated the basis for PNA reactivity of GC B cells. We found that GC B cell binding to PNA is associated with downregulation of the α2,3 sialyltransferase, *ST3GAL1* (ST3Gal1), and overexpression of ST3Gal1 was sufficient to reverse PNA binding in B cell lines. Moreover, we found that the primary scaffold for PNA-reactive O-glycans in B cells is the B cell receptor-associated receptor-type tyrosine phosphatase CD45, suggesting a role for altered O-glycosylation in antigen receptor signaling. Consistent with similar reports in T cells, ST3Gal1 overexpression in B cells *in vitro* induced drastic shortening in O-glycans, which we confirmed by both antibody staining and mass spectrometric O-glycomic analysis. Unexpectedly, ST3Gal1-induced changes in O-glycan length also correlated with altered binding of two glycosylation-sensitive CD45 antibodies, RA3-6B2 (more commonly called B220) and MEM55, which (in humans) have previously been reported to favor binding to naïve/GC subsets and memory/plasmablast subsets, respectively. Analysis of primary B cell binding to B220, MEM55, and several plant lectins suggested that B cell differentiation is accompanied by significant loss of O-glycan complexity, including loss of extended Core 2 O-glycans. To our surprise, decreased O-glycan length from naïve to post-GC fates best correlated not with ST3Gal1, but rather downregulation of the Core 2 branching enzyme GCNT1. Thus, our data suggest that O-glycan remodeling is a feature of B cell differentiation, dually regulated by ST3Gal1 and GCNT1, that ultimately results in expression of distinct O-glycosylation states/CD45 glycoforms at each stage of B cell differentiation.

## Introduction

B lymphocytes are essential mediators of prophylactic immunity, conferring durable immune protection through the secretion of soluble antigen-binding receptors called antibodies. The most effective B cell responses arise from the germinal center (GC) reaction, named for the transient microanatomical structures that appear in B cell follicles during B cell immune responses (1). The GC reaction is initiated by B cell activation by cognate T cells at the T-B follicular border, which leads to upregulation of the GC transcriptional program and T-B cell co-migration back into the B cell follicle. Within GCs, GC B cells undergo massive clonal expansion, somatically mutate their antibody binding sites, and undergo Darwinian-like selection for the highest affinity clones (1). After several rounds of proliferation and selection, GC B cells differentiate and exit the GC as either long-lived memory B cells or antibody secreting cells, both of which mediate pathogen clearance and provide durable prophylactic immunity against secondary antigenic encounter. However, this process is not infallible, and can result in poorly neutralizing antibodies, aberrant self-directed antibodies, or malignant transformation (1). Therefore, the continued unraveling of the mechanisms guiding GC responses remains a high priority for developing therapeutics that enhance or quell B cell responses in a variety of clinical settings, including generation of more potent vaccines.

A longstanding but still poorly understood aspect of GC B cells is GC reactivity with peanut agglutinin (PNA) (2, 3). PNA is a plant-derived glycan-binding protein (lectin) that exhibits strong binding to the serine/threonine (O)-linked disaccharide Gal β1,3-GalNAc-Ser/Thr, often referred to as Thomsen Friendenreich antigen or T-antigen, as well as extended O-glycan structures containing T-antigen (4–6). Typically, T-antigen is not exposed on healthy cells due to the further elaboration by other Golgi-resident glycosylation enzymes (glycosyltransferases) that modify the core T-antigen structure (7, 8). Indeed, T-antigen expression and associated PNA binding is a feature of malignant transformation (7, 8). Nonetheless, under rare circumstances, the T-antigen moiety can become transiently exposed in healthy cells. In T cells, T-antigen is expressed at specific stages of thymic development and after mature T cell activation and differentiation (2, 9–16). Functionally, altered T-antigen expression on the T cell coreceptor CD8 induces conformational changes that regulate CD8 affinity for MHC Class I, an interaction central to thymocyte positive selection (17, 18). Additionally, T-antigen expression on thymocytes and activated T cells is associated with synthesis of Core 2 poly-N-acetyllactosamines (poly-LacNAcs) (9–11, 19–25). These poly-LacNAcs regulate binding of immunoregulatory lectins known as galectins, which modulate thymocyte survival, mature T cell differentiation, and T cell effector function (25, 26). Simultaneously, Core 2 poly-LacNAcs also serve as scaffolds for synthesis of glycan functional groups such as sialyl lewis X, which drives selectin-mediated trafficking of T cells to tissues (27). Thus, altered PNA binding often heralds alterations to glycosylation that have important physiological consequences. Yet, despite being first reported almost 40 years ago (2, 3), the mechanisms and functional significance of PNA ligand exposure in GC B cells has remained unclear.

Here, we investigated the mechanisms underlying PNA binding, and attempted to generate insight into the function of this glycobiological change by identifying the scaffolds bearing PNA-reactive glycans. We present evidence that strongly implicates the α2,3-sialyltransferase *ST3GAL1* (ST3Gal1) in regulating the PNA phenotype of human GC B cells, particularly through modification of O-glycans on CD45. In the course of this investigation, we unexpectedly discovered that O-glycan remodeling is in fact not restricted to B cells at the GC stage, but rather a more general feature of B cell differentiation. Specifically, we observed that B cell differentiation to memory and plasmablast fates is associated with truncation of O-glycan chains, particularly of Core 2 O-glycans. Loss of Core 2 O-glycans toggled binding between the glycoform-specific CD45 antibodies B220 and MEM55, suggesting that this glycosylation switch occurs to a significant extent on CD45. Interestingly, although ectopic expression of ST3Gal1 was sufficient to truncate O-glycans *in vitro*, we found that expression of the Core 2 O-glycan branching enzyme GCNT1 best correlated with O-glycan length in primary B cells. Therefore, considering both T-antigen expression in GC B cells and O-glycan truncation with B cell differentiation, we conclude that global O-glycan remodeling is a general feature of B cell differentiation that drives expression of discrete CD45 glycoforms among distinct B cell populations.

## Results

### GC B Cells Downregulate Expression of the Core 1 O-Glycan Sialyltransferase *ST3GAL1*

**Figure 1:**
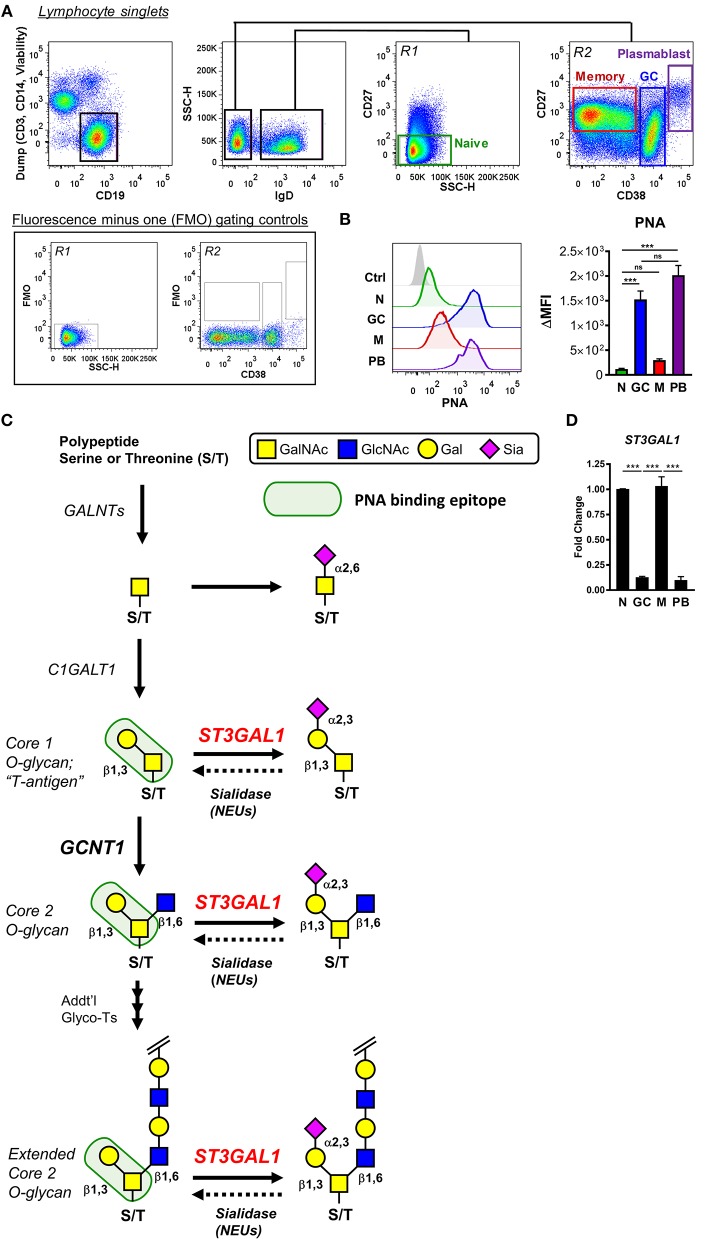
Expression of PNA-reactive glycans by germinal center B cells is correlated with downregulation of the α2,3-sialyltransferase ST3Gal1. **(A)** Gating strategy for analyzing tonsillar naïve (N), germinal center (GC), memory (M), and plasmablast (PB) B cells by flow cytometry. Representative fluorescence minus one (FMO) controls used for gating of CD27 are shown. **(B)** Analysis of peanut lectin binding to human tonsil B cells. Representative histograms of results are shown (*left*) as well as quantification of geometric mean fluorescence intensities (MFI) (*right*). **(C)** Schematic of synthesis of potential PNA-reactive O-linked glycans on B cells. O-glycan synthesis is initiated in the Golgi apparatus by polypeptide N-acetylgalactosamine transferases (*GALNTs*), which transfer a single GalNAc to select serine/threonine residues of a polypeptide backbone. The initiating GalNAc can be terminally sialylated, or further extended by C1GalT1 (*C1GALT1*) to form the simplest PNA-reactive epitope, a Core 1 O-glycan termed “T-antigen.” This core T-antigen moiety can be branched and elongated by other glycosyltransferases to form extended Core 2 O-glycans, which retain binding to PNA, or modified with sialic acid by the α2,3-sialyltransferase ST3Gal1 (*ST3GAL1*), which destroys PNA reactivity. Endogenous (or exogenous) sialidases may remove sialic acids and restore PNA binding. **(D)** Analysis of *ST3GAL1* expression in tonsillar B cells by quantitative real-time reverse transcription PCR (qRT-PCR), sorted as in **(A)**. Data are normalized to the housekeeping gene *VCP* and presented relative to naïve B cells. Data are representative of eight **(B)** or three **(D)** distinct tonsil specimens pooled from two **(B)** or three **(D)** independent experiments. Statistics were calculated using a Kruskal–Wallis test with Dunn's multiple comparisons test **(B)** or One-way analysis of variance (ANOVA) and Tukey's multiple comparisons test. Throughout, bars and error bars depict the mean and SEM, respectively. ns = not significant, ^***^p ≤ 0.001. ΔMFI, background subtracted geometric mean fluorescence intensity; GalNAc, N-acetylgalactosamine; Gal, galactose; Sia, sialic acid.

Palatine tonsils are sentinel lymphoid tissues continually exposed to oral microbes, and therefore represent a valuable and accessible site for study of human B cells. Using tonsil tissue discarded from routine tonsillectomies, we analyzed PNA binding to several B cell subsets *ex vivo*, including naïve, GC, memory, and plasmablast B cells (Figures 1A,B). As expected, GC B cells showed exceedingly strong binding to PNA that was >10-fold higher than naïve or memory B cells, indicating strong expression of O-glycans containing the asialylated Core 1 O-glycan moiety (T-antigen). Surprisingly, however, we found that plasmablasts also demonstrated strong binding to PNA that equaled that of GC B cells, suggesting that PNA reactivity may more accurately reflect B cell activation rather than be part of a GC program *per se*.

We reasoned that expression of T antigen or T-antigen-containing O-glycans (collectively, “PNA-reactive O-glycans”) in B cells may arise from one of several possibilities (Figure 1C). First, and most plausibly, PNA-reactive O-glycans may be expressed due to downregulation of sialyltransferases, which normally obstruct PNA binding by capping the galactosyl moiety of T-antigen with sialic acid. In this regard, the α2,3 sialyltransferase ST3Gal1 was the most plausible candidate due to its well-documented Core 1 O-glycan specificity and reported modulation of PNA binding in thymocytes and T cells (Figure 1C) (5, 12, 13, 19, 21, 28, 29). Second, expression and/or activity of sialic acid cleaving enzymes (sialidases) could also contribute to increased PNA binding by revealing T-antigen moieties. Third, augmented expression of PNA-reactive O-glycans in GC B cells may arise from increased expression of the T antigen-synthase glycosyltransferase, C1GALT1. Finally, an overall increased level of O-glycosylation could also potentially explain enhanced binding of PNA lectin (Figure 1C).

To narrow down which of these possibilities most likely accounted for increased expression of PNA-reactive O-glycans in GC B cells, we analyzed expression of O-glycosylation related genes among human naïve, GC, and memory B cells using publicly available expression array data (GSE12195) (30, 31). Analysis of O-glycosylation initiating enzymes, polypeptide N-acetylgalactosamine transferases (*GALNT*s) revealed no general upregulation of O-glycosylation in GC B cells that could account for increased T-antigen expression (Supplementary Figure 1). With the notable exception of *GALNT12* and *GALNT14*, expression of the vast majority of *GALNTs* were markedly downregulated in GC B cells, including *GALNT1, GALNT3, GALNT10, GALNT11, GALNT6* (compared to naïve), and *GALNT7* (compared to memory). Moreover, although T-antigen synthase (*C1GALT1)* and its essential chaperone Cosmc (*C1GALT1C1)* showed divergent expression, downregulation of *C1GALT1* in GC B cells suggests augmented Core 1 O-glycan synthesis is unlikely to account for increased T-antigen expression (Supplementary Figure 1). When sialidase expression was examined, we found that no endogenous sialidase genes (*NEU1-4)* were significantly upregulated in GC B cells compared to naïve or memory B cells. On the other hand, two sialyltransferase genes showed significantly decreased expression in GC B cells: *ST3GAL5* and *ST3GAL1*. Because *ST3GAL5* (also known as GM3 synthase) has been reported to predominantly act on lipids (32), *ST3GAL1* emerged as the most likely regulator of the PNA^hi^ phenotype of GC B cells.

Given that our preliminary microarray analysis implicated ST3Gal1, we next sought to validate this finding by quantitative real-time reverse transcription PCR (qRT-PCR). Indeed, flow cytometric sorting of primary tonsillar B cell subsets and qRT-PCR analysis revealed strikingly diminished *ST3GAL1* transcript levels in GC B cells and plasmablasts compared to naïve and memory B cells, in a manner reciprocal to PNA binding (Figure 1D). Therefore, these data supported diminished ST3Gal1 activity and loss of sialylation on Core 1 O-glycans as a major factor in expression of PNA-reactive O-glycans in primary GC B cells.

### ST3Gal1 Directly Modulates Expression of PNA-Reactive O-glycans in GC B Cells

**Figure 2:**
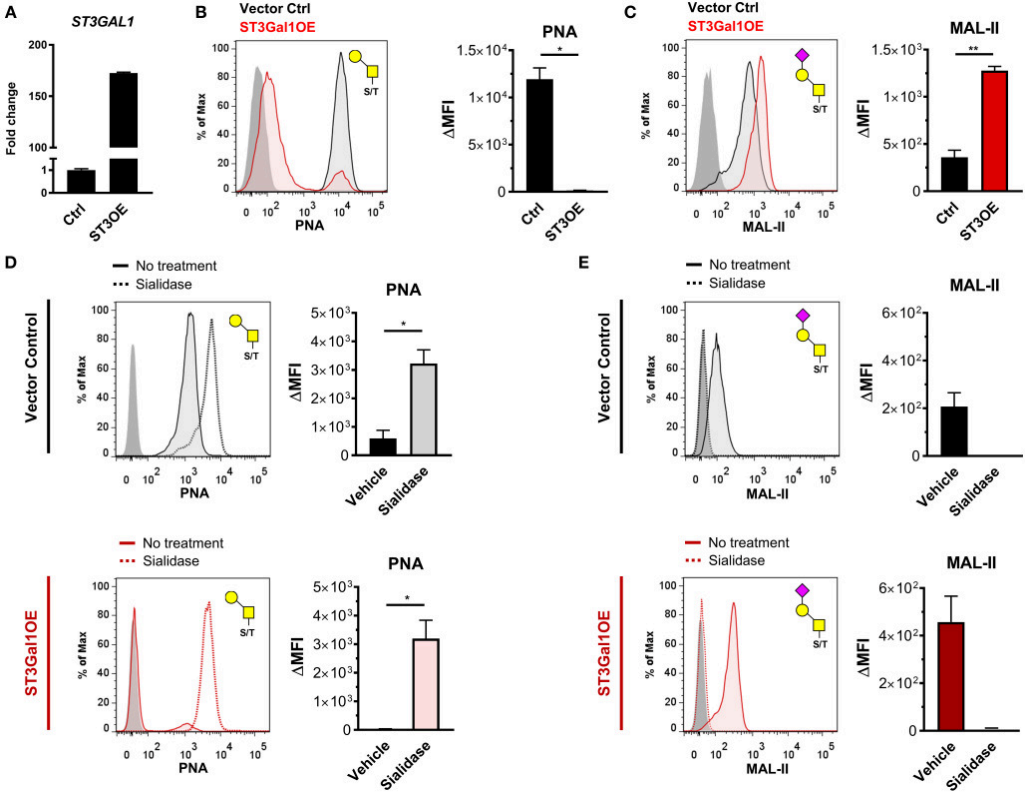
ST3Gal1 regulates PNA binding in B cells by sialylating Core 1 O-glycans. **(A)** Validation of *ST3GAL1* overexpression in Ramos B cells by qRT-PCR. Data were normalized to housekeeping control *VCP* and presented relative to vector control. **(B)** Representative histogram (*left*) and quantification (*right*) of flow cytometric analysis of PNA binding to vector control and ST3Gal1OE Ramos B cells. The Core 1 O-glycan/ T-antigen specificity of PNA is depicted at top right. **(C)** Representative histogram (*left*) and quantification (*right*) of flow cytometric analysis of MAL-II plant lectin binding to vector control and ST3Gal1OE Ramos B cells. The α2,3-sialylated Core 1 O-glycan/sialylated T-antigen glycan favored by MAL-II lectin is depicted at top right. **(D)** Representative histogram (*left*) and quantification (*right*) of PNA binding to vector control (*top*) or ST3Gal1OE Ramos B cells (*bottom)* before and after removal of sialic acids by intact cell treatment with *Arthrobacter ureafaciens* sialidase. **(E)** Representative histogram (*left*) and quantification (*right*) of MAL-II binding to vector control (*top*) or ST3Gal1OE (*bottom)* Ramos B cells before and after sialidase treatment, as in **(D)**. Data in **(B–E)** are from three independent experiments with three biological replicates in total. Statistics were calculated using Welch's unpaired, two-tailed *t*-test **(B–E)**. Throughout, bars and error bars depict the mean and SEM, respectively. ns = not significant, ^*^p ≤ 0.05, ^**^p < 0.01, ΔMFI, background subtracted geometric mean fluorescence intensity; Ctrl, vector control. ST3OE; ST3Gal1 overexpression.

To more directly test the hypothesis that ST3Gal1 regulates expression of PNA reactive O-glycans in B cells, we ectopically expressed ST3Gal1 in a PNA^hi^ GC-derived B cell line, Ramos (Figure 2A) (33), and analyzed the effect on PNA binding. Consistent with Core 1 O-glycan activity, ST3Gal1 overexpression (ST3Gal1OE) virtually ablated PNA binding entirely (Figure 2B) while augmenting binding to another plant lectin that preferentially binds α2,3-sialylated T-antigen, *Maackia amurensis* lectin-II (MAL-II) (Figure 2C) (34). By contrast, ST3Gal1OE had no significant effect on either complex N-glycan levels or α2,6-linked sialic acids, as measured by binding of *Phaseolus vulgaris* leucoagglutinin (PHA-L) and *Sambucus nigra* agglutinin (SNA), respectively (Supplementary Figure 2A). To validate whether this effect was specific to sialic acid and not due to off-target effects, we treated whole cells with *Arthrobacter ureafaciens* sialidase to remove sialic acids, and assessed the impact on PNA and MAL-II binding. As expected, sialidase treatment restored PNA binding to ST3Gal1OE B cells (Figure 2D). Sialidase treatment also augmented binding of PNA to control cells (empty vector transduced), suggesting that some sialylated Core 1 O-glycans were present even in PNA^hi^ Ramos cells. In all cases, sialidase treatment abolished binding of MAL-II lectin, consistent with the specificity of MAL-II for α2,3-sialylated Core 1 O-glycans (Figure 2E) (34). Taken together, these data strongly suggest that decreased levels of ST3Gal1 in primary GC B cells is a significant factor contributing to expression of PNA-reactive O-glycans in GC B cells.

### CD45 Is Major Scaffold Bearing PNA-Reactive Glycans in GC B Cells

**Figure 3:**
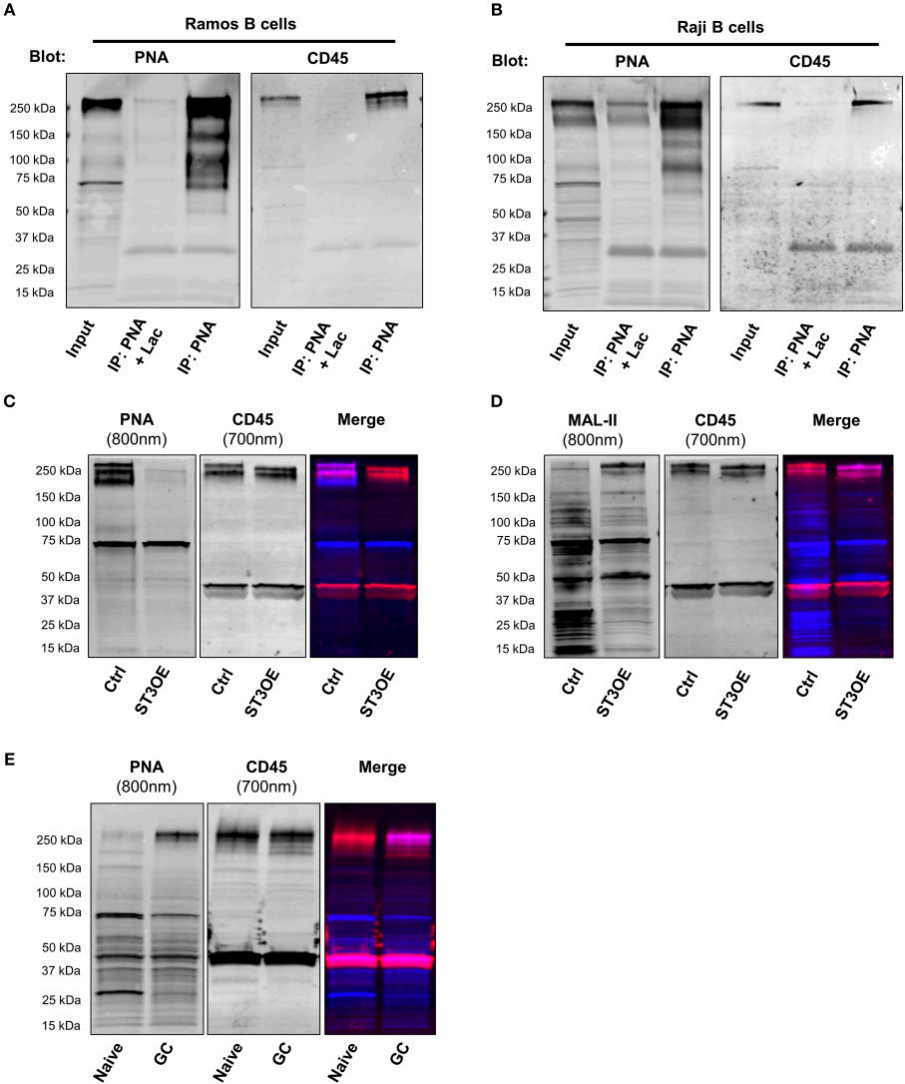
CD45 is a major glycoprotein bearing PNA-reactive O-glycans on B cells. **(A)** Immunoprecipitation (IP) with PNA-agarose beads from lysates of a GC-derived Burkitt lymphoma B cell line (Ramos), followed by SDS-PAGE and immunoblot with either PNA (*left*) or CD45 antibody (*right*). As a negative control, IP was also performed in the presence of a sugar inhibitor, lactose (Lac; middle lane). **(B)** IP and immunoblot of PNA-binding proteins of lysates from a second GC-derived Burkitt lymphoma B cell line (Raji), as in **(A)**. **(C)** Western blot analysis of PNA binding to Ramos vector control and ST3Gal1OE lysates (*left;* 800 nm fluorescence channel*)* followed by immunoblot with CD45 antibody (*middle*, 700 nm fluorescence channel). *Right*, merged. **(D)** Western blot analysis of staining of Ramos vector control and ST3Gal1OE lysates with MAL-II lectin (*left;* 800 nm fluorescence channel*)* followed by CD45 antibody (*middle*, 700 nm fluorescence channel). *Right*, merged. **(E)** Immunoblot of lysates from magnetically-enriched naïve and GC B cells with PNA (*left;* 800 nm fluorescence channel*)* followed by CD45 antibody (*middle*, 700 nm fluorescence channel). *Right*, merged. Data from **(A,B)** are from one experiment each showing similar results using three different B cell lines (Ramos, Raji, and SUDHL4; see also Supplementary Figure 2). Data in **(C–E)** are representative of three independent experiments with distinct cell aliquots or tonsil specimens. Ctrl, vector control; ST3OE, ST3Gal1OE.

In T cells, downregulation of ST3Gal1 and exposure of T-antigen or other PNA-reactive O-glycans is known to be correlated with activation, proliferation, enhanced T cell trafficking, increased susceptibility to cell death, and altered thymocyte selection (9, 12, 13, 17–20, 23, 35). In part, the diverse functions associated with PNA reactive glycans arise from both lectin-independent and lectin-dependent effects on multiple glycoprotein scaffolds, including CD8, CD43, and CD45 (9, 17–19, 21–23, 25, 36–38). Therefore, in order to better understand the functional significance of PNA-reactive O-glycans on B cells, we sought to identify the glycoproteins bearing these O-glycans. To this end, we immunoprecipitated PNA-binding glycoproteins from lysates of two PNA^hi^ B cell lines of purported GC origin, Ramos and Raji B cells, using PNA-agarose beads (33). Subsequent immunoblot with PNA (to maximize sensitivity) revealed several candidate bands, including a prominent ~260 kDa band in both Ramos and Raji lysates that was absent in negative control IP conditions (PNA-IP in the presence of lactose, a competitive inhibitor of PNA binding) (Figures 3A,B). Based on previous studies, we postulated that the 260 kDa band might correspond with CD45, which on B cells is expressed as a full-length isoform (“CD45RABC”) containing approximately 60 predicted O-glycosylation sites [NetOGlyc 4.0, http://www.cbs.dtu.dk/services/NetOGlyc/ (39)] predominantly clustered in exons 4, 5, and 6 (corresponding with A, B, and C isoforms) (25). Indeed, blotting with CD45 antibody revealed CD45 in PNA immunoprecipitates of both cell lines (Figures 3A,B). Notably, several other candidate bands at ~130 and 95 kDa were also revealed by PNA blot in Raji and Ramos B cells, although the identity of these bands was not determined. Preliminary mass spectrometric analysis of a gel fragment containing immunoprecipitated proteins >37 kDa revealed many other potential candidates besides CD45, including CD43 (~130 kDa when decorated with Core 2 O-glycans) and transferrin receptor (CD71, ~95 kDa in fully glycosylated form) (Supplementary Information 1), both of which have been reported to be modified with T-antigen in other cell types (25, 40). Because Ramos and Raji are both of Burkitt's lymphoma origin, we confirmed CD45/PNA co-immunoprecipitation in a third cell line, SUDHL-4, which is derived from a GC-type diffuse large B cell lymphoma (Supplementary Figure 2B) (33). Additionally, to rule out potential contribution of glycolipids to PNA binding, we analyzed PNA binding in Ramos B cells treated with the glucosylceramide synthase inhibitor D, 1-threo-phenyl-2-hexadecanoylamino-3-pyrrolidino-1-propanol (PPPP) (a gift from Dr. Ronald Schnaar, Johns Hopkins University). As expected, PPPP treatment showed very little effect on PNA binding, despite significant loss of the GC-enriched glycolipid Gb3 (CD77) (Supplementary Figure 2C).

Next, to test whether CD45 is a direct target of ST3Gal1, we analyzed PNA binding by lectin blot of ST3Gal1OE and control Ramos B cell lysates. Whereas control B cell lysates showed robust binding to PNA, overexpression of ST3Gal1 in Ramos B cells resulted in significantly diminished PNA binding, particularly of a band co-migrating with CD45 (Figure 3C). By contrast, the reverse binding pattern was observed in MAL-II lectin blots (Figure 3D).

To extend our analysis to primary B cells, we also examined potential PNA binding proteins present in tonsillar naïve and GC B cells. To this end, we magnetically enriched tonsillar naïve and GC B cells to >85% purity by positive selection with anti-IgD and anti-CD77 antigens, respectively, and then performed SDS-PAGE and PNA lectin blotting. In a similar manner to our findings in B cell lines, PNA blot revealed an analogous ~260 kDa band in GC lysates that was only faintly visible in naïve B cells (Figure 3E). Sequential probing with anti-CD45 antibody in a separate fluorescence channel revealed considerable co-migration between the ~260 kDa PNA-reactive band and CD45 (Figure 3E). Interestingly, the ~95 and ~130 kDa PNA-reactive bands observed in Ramos and Raji B cells were not apparent in either primary GC B cells or SUDHL-4 cells (Supplementary Figure 2B), suggesting that these proteins may bear PNA-reactive O-glycans in Burkitt lymphoma cells only. Thus, these data strongly suggest that CD45 is decorated by PNA-reactive O-glycans in GC B cells, and that the PNA-reactive T-antigen epitopes in these O-glycans are normally masked by ST3Gal1-mediated sialylation in naïve B cells.

### Ectopic Expression of ST3Gal1 Toggles Reactivity Between Glycoform-Specific CD45 Antibodies in a Manner Not Reversible by Sialidase

Our data suggested that non-GC and GC B cells express different CD45 glycoforms containing sialylated vs. asialylated Core 1 epitopes. Previous reports examining CD45 monoclonal antibody (mAb) binding between disparate B cell subsets have identified two CD45 mAb clones, RA3-6B2 (more commonly referred to as “B220”) and MEM55, that are sensitive to CD45 O-glycosylation and sialylation (41–44). (Note: While clone B220 is largely pan-reactive for B cells in mouse, it shows a more restricted binding within the human B cell pool). In particular, B220 binding has been shown to be enhanced by loss of sialic acid, whereas MEM55 binding has been shown to be absolutely dependent on sialic acid (41, 42, 44). We therefore reasoned that ST3Gal1-driven alterations to Core 1 sialylation on CD45 O-glycans might toggle expression of B220- and MEM55-reactive glycoforms.

**Figure 4:**
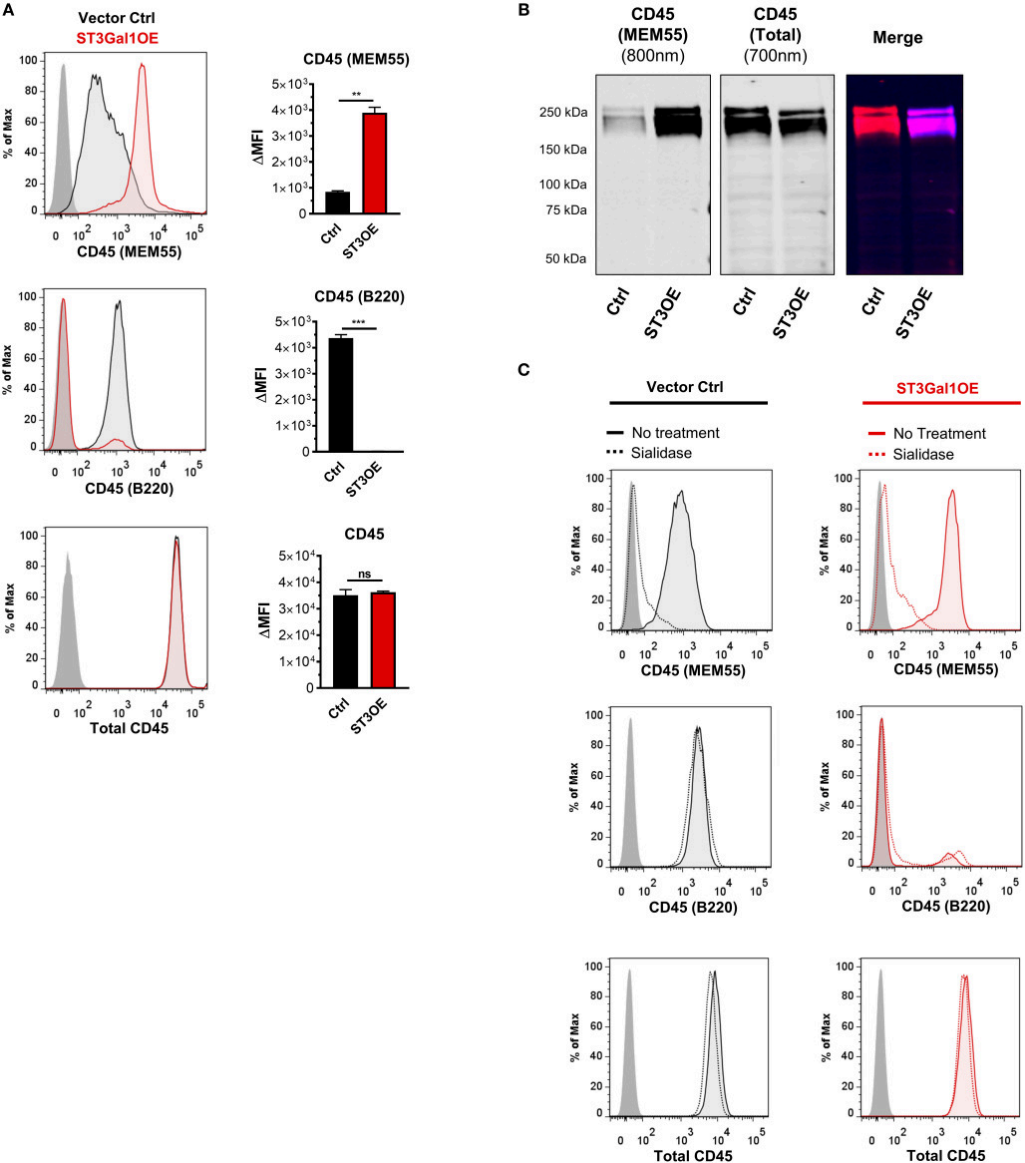
Overexpression of ST3Gal1 in B cells modulates binding of glycosylation sensitive CD45 antibodies. **(A)** Representative histogram (*left*) and quantification (*right*) of binding of two glycosylation-sensitive CD45 antibodies (MEM55 and B220) and one glycosylation-insensitive antibody (total CD45, clone HI30) to vector control and ST3Gal1OE Ramos B cells by flow cytometry. **(B)** Western blot analysis of binding of CD45 mAb MEM55 (800 nm fluorescence channel) and CD45 mAb HI30 (700 nm fluorescence channel) to Ramos vector control and ST3Gal1OE lysates. **(C)** Representative histograms depicting flow cytometric analysis of CD45 antibody binding to vector control and ST3Gal1OE Ramos B cells, before and after treatment with *Arthrobacter ureafaciens* sialidase. Data in **(A,C)** are from three independent experiments with three biological replicates. Data in **(B,C)** are representative of similar results from three **(B)** or two **(C)** independent experiments. Statistics in **(A)** were calculated using Welch's unpaired, two-tailed *t*-test. Throughout, bars and error bars depict the mean and SEM, respectively. ns, not significant, ^**^p ≤ 0.01, ^***^p ≤ 0.001, ΔMFI, background subtracted geometric mean fluorescence intensity; Ctrl, vector control; ST3OE, ST3Gal1OE.

To test this, we assayed binding of B220 and MEM55 CD45 mAbs to vector control and ST3Gal1OE Ramos B cells by flow cytometry. Strikingly, whereas control-transduced PNA^hi^ Ramos B cells displayed strong binding to B220 but not MEM55 mAb, overexpression of ST3Gal1 induced a reversal of mAb binding (Figure 4A). Western blot analysis of MEM55 binding to vector control and ST3Gal1OE Ramos B cells showed similar results and confirmed the CD45 specificity of this mAb (Figure 4B). Importantly, overexpression of ST3Gal1 did not affect binding of a glycosylation-insensitive CD45 mAb (HI30) (Figures 4A,B).

We next sought to test whether altered binding between B220 and MEM55 mAbs in ST3Gal1OE B cells was absolutely dependent on sialic acid. To test this, we treated B cells with *A. ureafaciens* sialidase to reverse ST3Gal1-mediated sialylation at the cell surface. Paradoxically, whereas MEM55 binding was completely dependent on sialic acid, as expected, cleavage of sialic acids in ST3Gal1OE B cells failed to restore binding of B220 to control levels (Figure 4C). Surprisingly, in contrast to previous reports, we did not observe enhanced binding of B220 in sialidase-treated Ramos vector control or ST3Gal1OE Ramos B cells. This result was unexpected and clearly did not fit a model in which sialylation was the only factor regulating binding between B220- and MEM55-reactive CD45 glycoforms. Thus, while ST3Gal1OE regulates binding of glycosylation-sensitive CD45 mAbs, loss of B220 binding could not be explained solely by the addition of a sialic acid moiety by ST3Gal1.

### ST3Gal1 Overexpression Blocks Formation of Core 2 O-glycans *in vitro*

**Figure 5:**
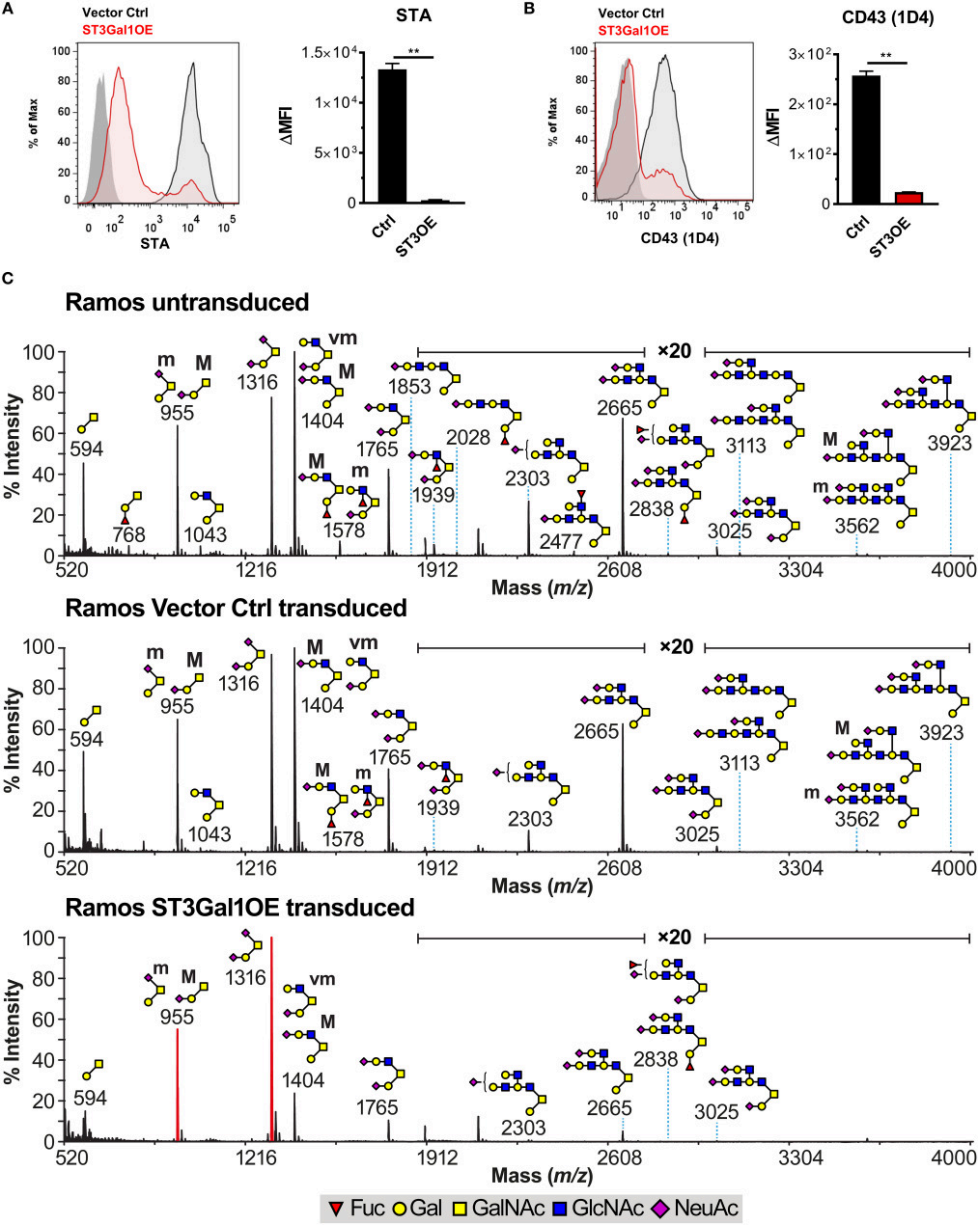
Overexpression of ST3Gal1 truncates O-glycans in B cells. **(A)** Representative histograms (*left*) and quantification (*right*) of binding of the Core 2 poly-LacNAc binding lectin *Solanum tuberosum* agglutinin (STA) to vector control and ST3Gal1OE Ramos B cells by flow cytometry. **(B)** Representative histograms (*left*) and quantification (*right*) of binding of the CD43 mAb 1D4 (Core 2 O-glycan-specific glycoform) to vector control and ST3Gal1OE Ramos B cells by flow cytometry. **(C)** Cellular O-glycome Reporter/Analysis (CORA) of untransduced Ramos, Ramos vector control, and Ramos ST3Gal1OE B cells. Depicted are MALDI-TOF MS spectra of peracetylated Benzyl-α-GalNAc-linked O-glycans. Structures above a bracket have not been unequivocally defined. Indicated areas in the spectra have a 20-fold magnification. “M” and “m” designations indicate major and minor abundances, respectively. Cartoon structures were drawn according to http://www.functionalglycomics.org guidelines and are representative from repeat experiments on two different biological replicates. Structure assignments are based on composition, tandem mass spectrometry, and biosynthetic knowledge. Full methods for MS analysis can be found in Materials and Methods. Data depict results from three **(A,B)** or two **(C)** biological replicates. Statistics in **(A)** and **(B)** were calculated using Welch's unpaired, two-tailed *t*-test. Throughout, bars and error bars depict the mean and SEM, respectively. ^**^p ≤ 0.01, ΔMFI, background subtracted geometric mean fluorescence intensity. Ctrl, vector control; ST3OE, ST3Gal1OE; Fuc, fucose; Man, mannose; Gal, galactose; GlcNAc, N-acetylglucosamine; GalNAc, N-acetylgalactosamine; NeuAc, N-acetylneuraminic acid (sialic acid).

The ability of ectopically expressed ST3Gal1 to regulate B220- and MEM55-associated CD45 glycoform expression in a non-reversible manner was surprising, and did not comport with a purely sialic acid-dependent mechanism of action. Rather, the non-reversible nature of this effect implied other structural changes to CD45 O-glycans that could not be reversed at the cell surface by treatment with exogenous sialidase. Besides preferred sialylation of T-antigen, ST3Gal1 has also been reported to block Core 2 O-glycan formation by competing with the Core 2-branching enzyme GCNT1 for the T-antigen precursor (Figure 1C) (19–21). Additionally, it has previously been reported that B220 binding correlates with expression of Core 2 O-glycans (44). We therefore reasoned that loss of Core 2 O-glycans due to competition with GCNT1 may account for impaired B220 binding (and resulting gain of MEM55 binding) in ST3Gal1OE B cells. To test this, we analyzed binding of two Core 2 O-glycan-reactive reagents: *Solanum tuberosum* agglutinin (STA), which binds poly-LacNAc on Core 2 O-glycans (and also poly-LacNAc on N-glycans); and the CD43 mAb 1D4, which specifically binds CD43 modified with elongated Core 2 O-glycans (45). Consistent with a competitive role for ST3Gal1, binding of STA and 1D4 were both drastically reduced in ST3Gal1OE B cells (Figures 5A,B), suggesting that high expression of ST3Gal1 is sufficient to block Core 2 O-glycan formation by GCNT1 in B cells.

To more precisely define the B cell glycan repertoire associated with B220 and MEM55 binding, we analyzed the N- and O-linked glycomes of untransduced, vector control transduced, and ST3Gal1OE Ramos B cells by mass spectrometry (MS). For analysis of O-glycomes, we utilized both conventional O-glycomics MS techniques as well a recently developed highly sensitive technique known as Cellular O-glycome Reporter Amplification (CORA) (46, 47). Using both approaches, we observed significant structural alterations between control and ST3Gal1OE B cells, including an expected increase in the ratio of sialylated to asialylated Core 1 O-glycan structures in ST3Gal1OE B cells (Figure 5C; Supplementary Figure 3). However, consistent with a competitive relationship between ST3Gal1 and GCNT1 (at least when ST3Gal1 is expressed at very high levels), ST3Gal1OE B cells exhibited a striking reduction in Core 2 O-glycans compared to controls (Figure 5C; Supplementary Figure 3). Intriguingly, we also noted I blood group antigen (“I-branch”) expression on Core 2 O-glycan poly-LacNAcs in untransduced and vector control Ramos B cells that was particularly apparent in samples prepared by the CORA technique (Figure 5C). I-branch expression on N-glycan poly-LacNAcs has recently been shown by our laboratory to be a feature of GC B cells (48), and because Ramos B cells are believed to have arisen from a lymphoma of GC origin (Burkitt's lymphoma) (33), these data suggested that GC B cells may also express I-branches on poly-LacNAcs of O-glycans.

Subsequent analysis of the N-glycomes of untransduced, control, and ST3Gal1OE Ramos B cells revealed largely similar results in all three groups, suggesting that ST3Gal1 predominantly acts on O-glycans, as reported. Specifically, we found that all three groups uniformly expressed high levels of multi-antennary complex N-glycans modified with I-branched poly-LacNAcs (Supplementary Figure 4), in a manner highly concordant with the N-glycomic phenotype of primary GC B cells (48). However, while the N-glycomes were mostly unperturbed by ST3Gal1, we did note slightly reduced quantities of I-branches on N-glycans in ST3Gal1OE B cells, possibly resulting from the increased metabolic demand imposed by ST3Gal1 overexpression (Supplementary Figure 4).

Taken together, these data suggest that B220 mAb binding to CD45 is associated with B cell expression of elongated Core 2 O-glycans, whereas MEM55 binding to CD45 is associated with B cell expression of predominantly truncated Core 1 O-glycans. Moreover, whereas untransduced and control-transduced Ramos B cells natively express B220^hi^ and PNA^hi^ CD45 glycoforms, overexpression of ST3Gal1 *in vitro* is sufficient to convert CD45 to MEM55^hi^ PNA^lo^ glycoforms.

### B Cell Differentiation Is Associated With Progressive Loss of O-glycan Complexity

In previous studies, naïve and GC B cells were found to preferentially express B220-reactive glycoforms of CD45, whereas memory and plasmablast subsets preferentially expressed MEM55-reactive glycoforms (41–43). In the present study, we observed that B220 mAb binding corresponded with global expression of elongated Core 2 O-glycans, whereas MEM55 mAb binding corresponded with expression of sialylated, truncated O-glycans. Therefore, we reasoned that differences in binding between B220- and MEM55-reactive primary B cells may thus also correspond with differences in O-glycosylation.

**Figure 6:**
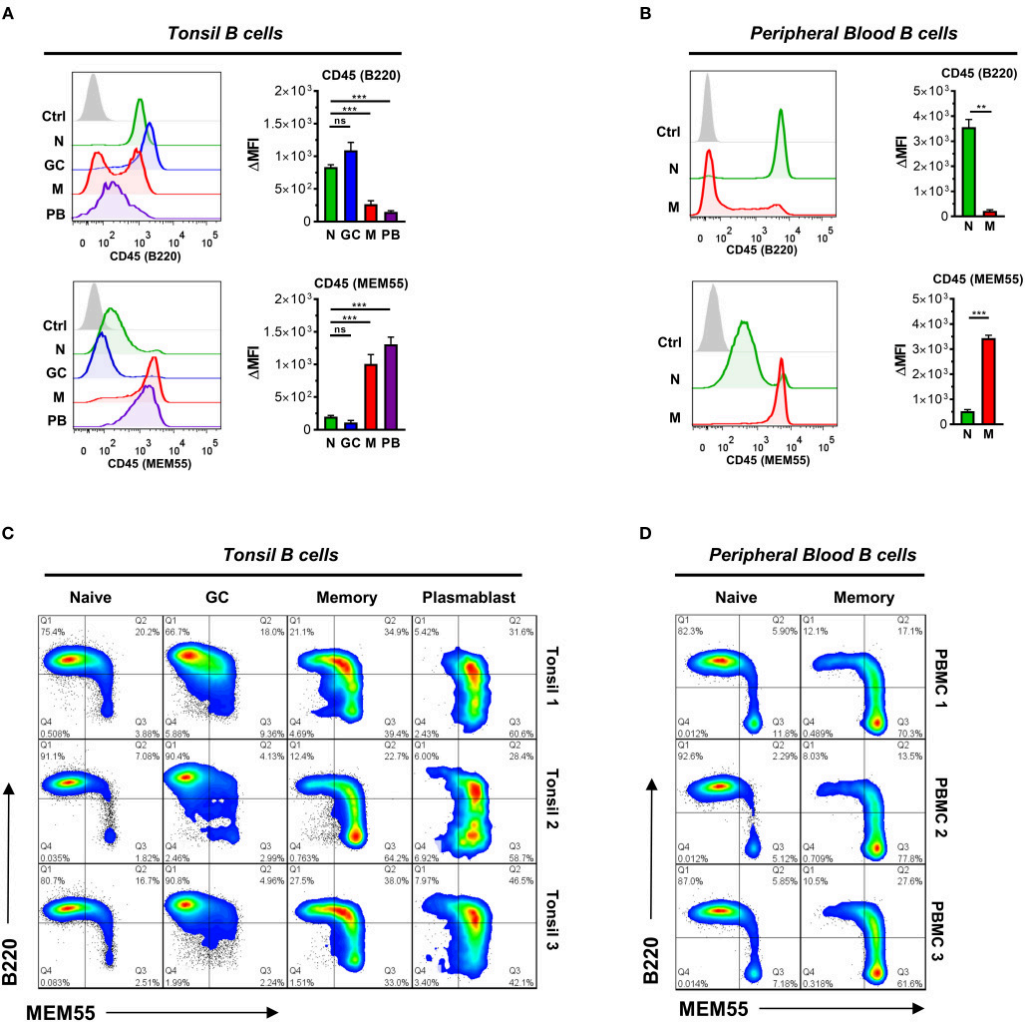
B cells transition from B220- to MEM55-reactive CD45 glycoforms during B cell differentiation. **(A)** Representative histograms (*left*) and quantification (*right*) of binding of glycosylation-sensitive CD45 antibodies B220 and MEM55 to the indicated tonsillar B cell subset. Subsets are gated as in Figure 1A. **(B)** Representative histograms (*left*) and quantification (*right*) of binding of B220 and MEM55 to peripheral blood naïve (CD19+ IgD+ CD27-) and memory (CD19+ IgD- CD27+) B cells. **(C)** Pseudocolored smoothed dot plots depicting binding of MEM55 and B220 to tonsillar naïve, GC, memory, and plasmablast B cells in three distinct tonsil specimens. **(D)** Pseudocolored smoothed dot plots depicting binding of MEM55 and B220 to peripheral blood naïve and memory B cells in three distinct peripheral blood donor specimens. For **(A,B)**, *n* = 5 and *n* = 3, respectively. For **(C,D)**, data depict three separate tonsil and peripheral blood specimens, respectively. Statistics were calculated using a Kruskal–Wallis test with Dunn's multiple comparisons test **(A)** or an unpaired, two-tailed Welch's *t*-test **(B)**. Throughout, bars and error bars depict the mean and SEM, respectively. ns, not significant, ^**^p ≤ 0.01, ^***^p ≤ 0.001. ΔMFI, background subtracted geometric mean fluorescence intensity.

To test this, we first examined binding of B220 and MEM55 to tonsillar and peripheral blood B cells by flow cytometry. As reported, B220 showed preferential binding to naïve and GC B cells (42, 43), whereas MEM55 exhibited superior binding to memory B cells and plasmablasts (Figures 6A,B) (41). We noted significant heterogeneity in binding, particularly among the memory B cell population. Therefore, to better dissect expression of B220 and MEM55 among B cell populations, we implemented a dual B220/MEM55 staining approach to assess whether expression of B220/MEM55 glycoforms was mutually exclusive or able to be co-expressed. Intriguingly, we found that while memory and plasmablasts showed clear bias toward MEM55 expression over B220 expression, a significant portion of memory and plasmablasts appeared to be in transition and exhibited binding to both antibodies (Figure 6C). Dual mAb binding was less apparent in peripheral blood B cells (Figure 6D), suggesting that CD45 glycosylation may be actively remodeled during ongoing immune responses (such as in tonsil) but may be more stable in resting B cell populations (such as in peripheral blood of healthy donors). Whereas we did not note enhancement of B220 binding in sialidase-treated Ramos B cell lines (Figure 4C), treatment of primary tonsillar B cells with *A. ureafaciens* sialidase did slightly enhance B220 binding; MEM55 binding, by contrast, was almost completely abolished by sialidase treatment, as previously reported (Supplementary Figure 5B) (41, 42, 44). Although GC B cells did display a slightly higher amount of CD45, significant differences in total CD45 levels were insufficient to explain differences in binding of either antibody between B cell subsets (Supplementary Figure 5A).

**Figure 7:**
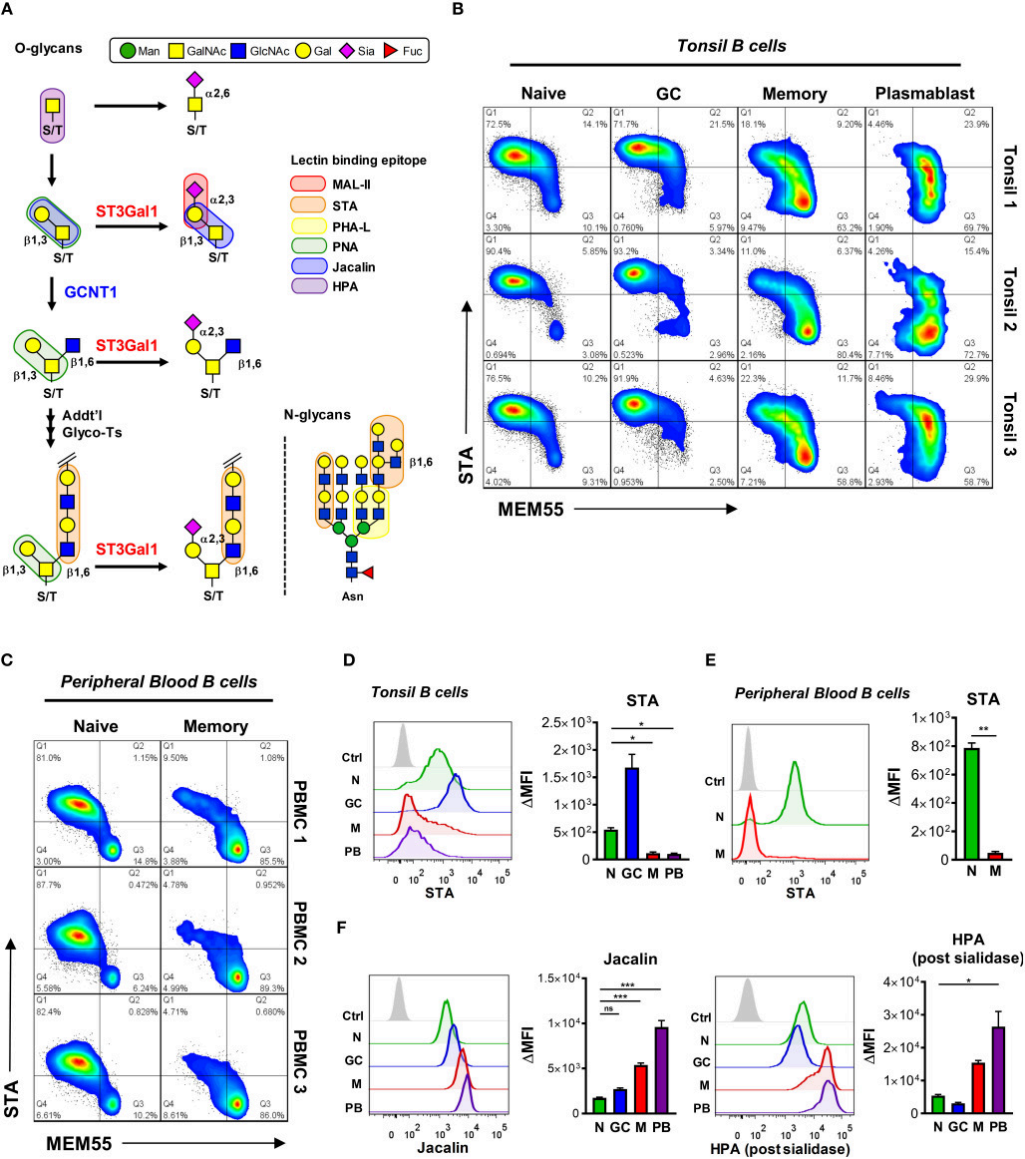
Primary B cell conversion from B220- to MEM55-reactive CD45 glycoforms is associated with truncation of O-glycans. **(A)** Schematic of O-glycan biosynthesis and corresponding plant lectin binding epitopes. A representative N-glycan is also shown to illustrate N-glycan specificities, where applicable. **(B)** Pseudocolored dot plots depicting dual staining of MEM55 mAb and STA plant lectin in tonsillar B cell subsets from three distinct tonsil specimens. **(C)** Pseudocolored dot plots depicting dual staining of MEM55 mAb and STA plant lectin in peripheral blood naïve (CD19+ IgD+ CD27−) and memory (CD19+ IgD− CD27+) B cell subsets from three distinct healthy donors. **(D)** Representative histograms *(left)* and quantification *(right)* of binding of STA plant lectin to tonsillar B cells by flow cytometry. **(E)** Representative histograms *(left)* and quantification *(right)* of binding of STA lectin to peripheral blood naïve and memory B cells by flow cytometry. **(F)** Representative histograms *(left)* and quantification *(right)* of binding of Jacalin and HPA plant lectins to primary tonsillar B cells by flow cytometry. For HPA staining, cells were first treated with Arthrobacter ureafaciens sialidase. Data depict three **(B,C,E)**, eight **(D)**, nine (**F**, Jacalin), or six (**F**, HPA) distinct tonsil specimens. Statistics were calculated using a Kruskal–Wallis test with Dunn's multiple comparisons test **(D,F)** or Welch's unpaired two-tailed T-test (E). Throughout, bars and error bars depict the mean and SEM, respectively. ns, not significant, ^*^p < 0.05, ^**^p < 0.01, ^***^p < 0.001. MFI, background subtracted geometric mean fluorescence intensity; N, naïve; GC, germinal center; M, memory; PB, plasmablast; Man, mannose; GalNAc, N-acetylgalactosamine; GlcNAc, N-acetylglucosamine; Gal, galactose; Sia, sialic acid; Fuc, fucose.

Based on our O-glycomic analysis of B cells expressing B220- and MEM55-reactive CD45 glycoforms *in vitro*, we hypothesized that transition of B220 to MEM55 binding in primary B cells would be associated with truncation of O-glycans. To test this, we analyzed binding of several O-glycan-reactive plant lectins, including STA, Jacalin, MAL-II, and *Helix pomatia* (HPA) lectins, to primary B cells. The binding preferences of each lectin is graphically depicted in Figure 7A (5). Consistent with our results *in vitro*, both tonsillar and peripheral blood B cells that acquired MEM55 reactivity showed significantly reduced binding to STA lectin, consistent with reduced expression of Core 2 O-glycan poly-LacNAcs (Figures 7B–E). Consistent with a recent study by our laboratory, we also observed much higher binding of STA to GC B cells compared to naïve B cells, attributable to differences in I-branching of N-glycans between naïve and GC B cells rather than differences in Core 2 O-glycan expression (48). Besides STA, Jacalin lectin [which binds sialylated and asialylated T-antigen, but not in the presence of Core 2 O-glycans (49)] and HPA lectin [which binds terminal GalNAc, especially truncated O-glycans consisting of a single GalNAc moiety (5, 50)] both showed dramatically enhanced binding to more differentiated B cells compared to naïve and GC B cells, suggesting a progressive decrease in O-glycan length with differentiation (Figure 7F). MAL-II lectin also showed starkly increased binding to more differentiated B cells compared to naïve and GC B cells, possibly reflecting an inability of this lectin to bind sialylated T-antigen modified by Core 2 O-glycans (Supplementary Figure 5C, *top***)**. By contrast, the N-glycan specific lectin PHA-L did not show similar trends as Jacalin or HPA lectins, suggesting that increased binding of these lectins was not simply due to increased cell size (Supplementary Figure 5C, *bottom***)**. Thus, these data suggest that B cell differentiation to memory B cell and plasmablast fates is associated with a general loss in O-glycan complexity. Moreover, when considered together with our O-glycomic analysis of B220 and MEM55-reactive Ramos B cells, our data suggest that the B220 to MEM55 conversion reflects a transition in CD45 glycoform expression from elongated, Core 2-containing (naïve and GC) to truncated and sialylated (memory and plasmablast).

### Reduced O-glycan Complexity With B Cell Differentiation Correlates With Decreased Expression of GCNT1

In our *in vitro* studies, we observed that overexpression of ST3Gal1 induced O-glycan truncation that converted B cells from B220- to MEM55-reactive (Figures 4, 5). However, in primary cells, expression of ST3Gal1 did not readily correlate with O-glycan chain length (Figures 1D, 6, 7). Indeed, naïve and memory B cells possessed similar transcript levels of *ST3GAL1*, despite exhibiting significant differences in O-glycan length. Therefore, these data suggest that truncation of O-glycans by ST3Gal1 may be highly dependent on level of expression, and (at least in B cells) may inhibit formation of Core 2 O-glycans only when expressed to a very high degree. In this regard, we reasoned that, in parallel with ST3Gal1-mediated sialylation of T-antigen, a second mechanism may be operating to regulate differences in O-glycan chain length with B cell differentiation.

**Figure 8:**
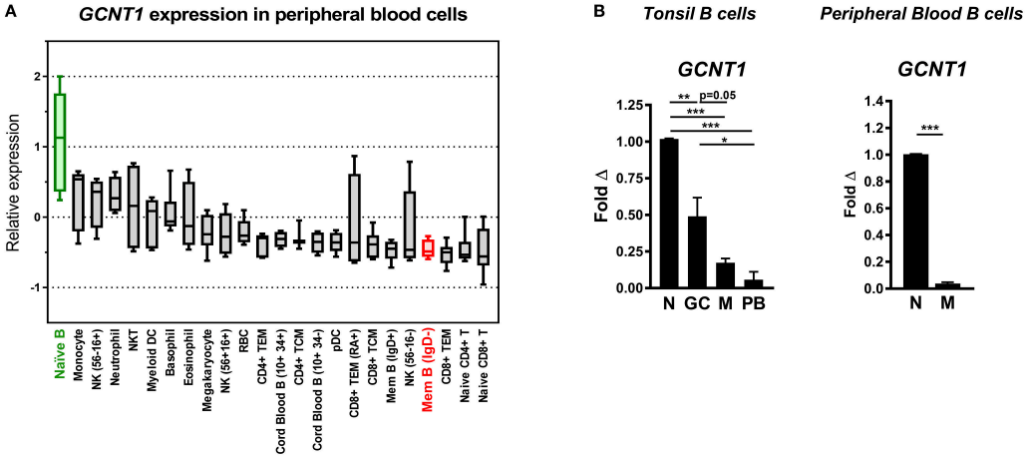
Loss of O-glycan complexity with differentiation is associated with downregulation of the Core 2 branching enzyme GCNT1. **(A)** Relative mean *GCNT1* expression in peripheral blood cells, analyzed from the publicly available dataset *GSE24759*. Box and whiskers depict minimum value, maximum value, median, and interquartile range. **(B)** qRT-PCR analysis of transcript levels of *GCNT1* in primary tonsil and peripheral blood B cell subsets. Data were normalized to the housekeeping gene *VCP* and are depicted relative to naïve B cells. For **(B)**, *n* = 3 distinct tonsil or peripheral blood specimens. Bars and error bars in **(B)** depict mean and SEM. ^*^p ≤ 0.05, ^**^p ≤ 0.01, ^***^p ≤ 0.001. N, naïve; GC, germinal center; M, memory; PB, plasmablast.

One possible explanation for the observed loss in O-glycan complexity with B cell differentiation is downregulation of the Core 2 branching enzyme GCNT1. As described earlier, GCNT1 initiates formation of Core 2 poly-LacNAc chains by transferring a GlcNAc moiety to a Core 1 O-glycan precursor (51, 52). Indeed, analysis of *GCNT1* expression in a publicly available dataset (53) revealed that naïve B cells, which are B220^hi^ (Figure 6), expressed the highest mean transcript levels of *GCNT1* of all hematopoietic subsets, whereas memory B cells, which are B220^lo^ and MEM55^hi^ (Figure 6), possessed among the lowest *GCNT1* transcript levels (Figure 8A). Subsequent sorting and qRT-PCR analysis of *GCNT1* expression in primary naïve, GC, memory, and plasmablast B cells confirmed that *GCNT1* was robustly expressed in naïve B cells but steadily declined with B cell differentiation (Figure 8B, *left*). Similar findings were observed with naïve and memory B cells from peripheral blood (Figure 8B, *right*). Therefore, these data strongly suggest that Core 2 O-glycans are expressed by less differentiated naïve and GC B cells due to high levels of GCNT1, but are strongly downregulated in more differentiated B cell subsets, including memory B cells and plasmablasts, due to diminished GCNT1 expression.

**Figure 9:**
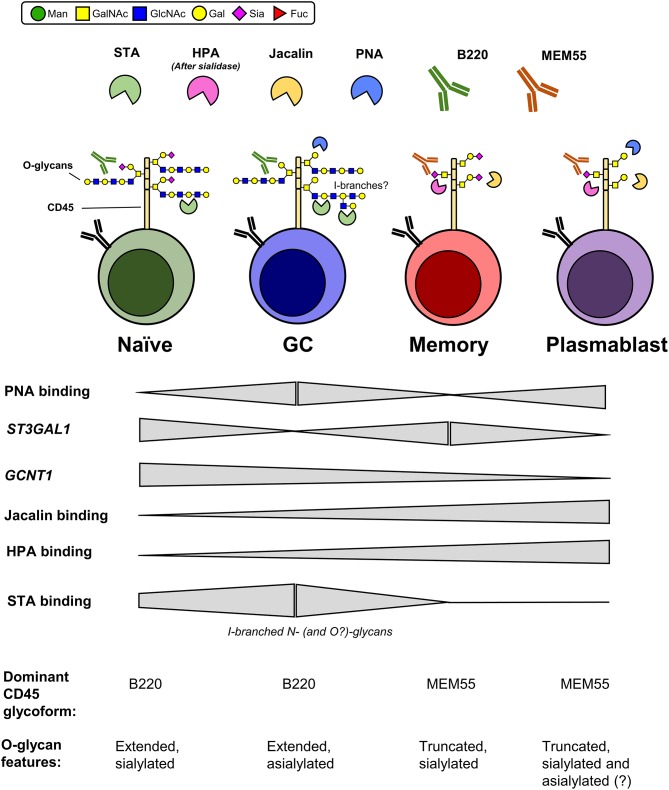
Proposed O-glycosylation phenotype of human B cell subsets and expression of associated CD45 glycoforms. Summary of relative plant lectin binding, glycosyltransferase gene expression, and expression of CD45 mAb-reactive glycoforms for each B cell subset, as determined in the present study. Glycan features that are inconclusive are marked with a “?”. Man, mannose; GalNAc, N-acetylgalactosamine; GlcNAc, N-acetylglucosamine; Gal, galactose; Sia, sialic acid; Fuc, fucose.

Taken together, our data support a model in which two O-glycosylation enzymes, ST3Gal1 and GCNT1, separately regulate two glycosylation features during B cell differentiation: α2,3-sialylation of Core 1 O-glycans and formation/extension of Core 2 O-glycans, respectively. Whereas naïve B cells exhibit sialylated, elongated O-glycans, our data suggest that these O-glycans become transiently unsialylated and then progressively shortened with GC- and post-GC differentiation, respectively. Additionally, in combination with results examining binding of CD45 mAbs B220 and MEM55, we further propose that these global alterations in glycosylation drive expression of distinct CD45 glycoforms at each stage of B cell differentiation (summarized in Figure 9).

## Discussion

The PNA binding phenotype of GC B cells was first described by Rose and colleagues nearly 40 years ago (2, 3). Surprisingly, however, the glycobiological mechanisms driving PNA reactivity, and its physiological significance, have remained unclear. Here, we investigated the glycobiological basis for PNA reactivity of GC B cells. We found that the most plausible explanation for PNA-reactivity of GC B is downregulation of ST3Gal1, a sialyltransferase with a reported preference for Core 1 O-glycans (5, 12, 13, 19, 21, 28, 29). Overexpression of ST3Gal1 was sufficient to ablate PNA binding to a GC-derived cell line, Ramos. Functionally, CD45, a central regulator of BCR signaling, emerged as a plausible scaffold for PNA-reactive glycans in primary GC B cells. We further found that ST3Gal1 overexpression unexpectedly toggled reactivity between two glycosylation-sensitive CD45 mAbs, B220 and MEM55, by truncating Core 2 O-glycans. Analysis of B220 and MEM55 binding in primary B cells, in conjugation with several O-glycan binding plant lectins, revealed a gradual transition during B cell differentiation from expression of extended Core 2 poly-LacNAc-containing O-glycans to shorter, sialylated O-glycans. In contrast to *in vitro* studies, ST3Gal1 in primary B cells did not readily correlate with O-glycan length but rather expression of the Core 2 branching enzyme GCNT1. Therefore, in the course of investigating PNA reactivity of GC B cells, we uncovered two distinct differentiation-associated alterations in O-glycosylation, both of which occur at least in part on the glycoprotein CD45 and are regulated in parallel by the O-glycosylation enzymes ST3Gal1 and GCNT1.

Our finding that ST3Gal1 modulates the PNA phenotype of GC B cells is not entirely unexpected, given similar reports for ST3Gal1 in modulating T-antigen expression (and related O-glycans) in thymocytes and mature T cells (2, 9–16). However, our study now provides evidence for a similar ST3Gal1-mediated mechanism in GC B cells that was perhaps suspected, but to our knowledge, not rigorously investigated. In our analysis of PNA binding, we also observed that plasmablasts were also strongly reactive with PNA and similarly downregulated ST3Gal1, suggesting that exposure of PNA-reactive O-glycans is a general feature of activated B cells and not part of a GC-specific program. However, somewhat puzzlingly, plasmablasts strongly bind both PNA (reactive with glycans containing asialylated T-antigen) and MAL-II lectins (reactive with sialylated T-antigen) compared to naïve B cells. This disparity might be explained if MAL-II lectin binding is inhibited by the presence of Core 2 O-glycosylation. Based on these data, the precise sialylation status of Core 1 O-glycans in plasmablasts is difficult to define.

In addition to PNA ligand exposure on GC B cells, several glycosylation-sensitive CD45 mAbs had previously been reported to differentially bind disparate B cell subsets (41–43). These data suggested that CD45 may transition through several glycoforms during B cell differentiation. However, the nature of these glycoforms have remained largely undefined, because the glycans associated with antibody binding had not been extensively analyzed. Our O-glycomic analysis of B220-reactive and MEM55-reactive B cells suggests that naïve and GC B cells (B220-reactive) express bulky glycoforms of CD45 containing Core 2 O-glycan poly-LacNAcs, whereas memory and plasmablast B cells (MEM55-reactive) express shorter and more sialylated glycoforms of CD45. Expression of Core 2 O-glycans primarily by naïve and GC B cells is supported by the higher levels of GCNT1 expressed by these subsets compared to memory and plasmablasts. Notably, this model is also supported by a recent O-glycomic study by Macauley and colleagues, in which the authors were able to detect Core 2 poly-LacNAcs on bulk tonsil B cells, which are composed primarily of naïve and GC B cells (54). The expression of Core 2 O-glycans and high levels of GCNT1 by naïve B cells is surprising and is opposite of from the expression pattern of T cells. Whereas naïve T cells express shorter O-glycans and upregulate Core 2-containing O-glycans with activation, B cells appear to exhibit the reverse behavior (9, 25). The functional significance of this difference will be an important area of future investigation.

An interesting observation arising from the disparate sialylation and O-glycosylation status of naïve, GC, memory, and plasmablasts is that each subset expresses a distinct CD45 glycoform at each stage of B cell differentiation (Figure 9). What might be the physiological significance of these distinct glycoforms? Ostensibly, modular glycosylation between B cell subsets might serve as an analogous mechanism to CD45 isoform switching on human T cells (25). In a study by Xu and Weiss, sialylation and O-glycosylation of CD45 were found to inhibit homodimerization-induced inactivation, thereby enhancing CD45 activity (55). By this model, differential sialylation and O-glycosylation of CD45 may serve to intrinsically tune CD45 signaling at different stages of B cell maturation. Besides intrinsic CD45 signaling, differential CD45 O-glycosylation may regulate interaction with endogenous lectins. Indeed, in studies using DLBCL B cells, Clark and colleagues found that Core 2 O-glycans, regulated by GCNT1, were required for optimal CD45 binding of galectin-3, which upon binding dampened CD45 phosphatase activity and promoted B cell survival (56). Moreover, studies assessing N-glycosylation of CD45 have also noted critical roles for differential N-glycosylation between B cell subsets in the regulation of galectin binding. For instance, our lab has recently reported that differential I-branching of N-glycans between resting and GC B cells is a major regulator of binding of the inhibitory lectin galectin-9, which in B cells dampens BCR calcium signaling (48). Besides galectins, the sialic acid-binding inhibitory receptor CD22 has also recently been shown to be regulated by glycan-dependent interactions with CD45 (57, 58), as well as altered GlcNAc sulfation between naïve/memory and GC B cells (54). Thus, alterations in O-glycosylation may serve analogous functions in the regulation of lectin binding in *cis* or in *trans*. Finally, alterations in glycosylation on CD45 may also serve to regulate intercellular communication, either through intrinsic properties (such as the negative charge of sialic acid) or through lectin-mediated interactions in *trans*. The expression of unique glycoforms of CD45 in different B cell subsets may therefore serve not only to differentially regulate CD45 activity, but also to dictate the strength and/or repertoire of lectin binding in *cis* and *trans*.

Taken together, our data suggest that B cells undergo extensive alterations in O-glycosylation with B cell differentiation that drive expression of distinct CD45 glycoforms. These findings add to a growing body of evidence indicating that lymphocytes undergo glycan remodeling in order to acquire or discard specific functionality at discrete stages of differentiation.

## Materials and Methods

### Contact for Reagent and Resource Sharing

Requests for reagents or additional information should be directed to corresponding author, Charles J. Dimitroff (cdimitroff@bwh.harvard.edu).

### Oligonucleotide Sequences

Primers and other oligonucleotide sequences used in this study can be found in Supplementary Table 1.

### Antibodies and Reagents

A full list of antibodies and reagents used in this study can be found in Supplementary Table 2.

## Cell Lines

Ramos and Raji cells were generously provided by Dr. Shiv Pillai (Ragon Institute of MGH, MIT, and Harvard). SUDHL-4 B cells were a gracious gift from Dr. Alan Epstein (USC Keck School of Medicine). All B cell lines were maintained at 0.5 × 10^6^-2.0 × 10^6^ cells mL^−1^ in complete RPMI medium [RPMI 1640 + 10% (v/v) FBS + 25 mM HEPES + 1% (v/v) Penicillin/Streptomycin]. Media was renewed every 2–3 days (Ramos, Raji) or every 3–5 days (SUDHL-4). For each cell line, aliquots were frozen in cell culture media supplemented with 10% FBS and stored in the vapor phase of a liquid nitrogen freezer for later use.

To generate ST3Gal1 overexpression Ramos B cells, human ST3Gal1 cDNA (Origene #SC111017) was amplified by PCR and then subcloned into pLVX-EF1α-IRES-ZsGreen1 (Clontech #631982), a bicistronic lentiviral expression vector allowing for simultaneous co-expression of ST3Gal1 and ZsGreen1 from a single mRNA transcript. The ST3Gal1 insert was sequenced and was found to match the NCBI reference sequence NM_173344.2 for ST3Gal1 transcript variant 2, except for one synonymous mutation at base 261 (C->T) of the coding sequence. Lentivirus containing the ST3Gal1 construct was produced by co-transfection of HEK293T cells with the helper plasmids pMD2.G-VSV-G and psPAX2-Δ8.9 using Lipofectamine 2000 (Thermo #11668-027). Forty-eight hours later, 1 × 10^6^ Ramos B cells were resuspended in 1 mL of viral supernatant, plated in 24 well flat-bottom tissue culture plates, and spinfected at 1,000 × g for 90 min at room temperature, followed by culture in fresh media for 24 h. Successfully transduced cells were sorted to >99% purity on ZsGreen1-fluorescent cells by flow cytometric sorting on a BD FACSAria at the Harvard Division of Immunology's Flow Cytometry Core. Sorted ST3Gal1-expressing (ZsGreen1+) cells were expanded and frozen for subsequent use.

### Expression Array Analysis

Raw Affymetrix HG-U133plus2 CEL file data for sorted tonsillar B cell subsets were downloaded from NCBI GEO (GSE12195) and dChip (59) was used to normalize probe hybridization intensities across arrays, followed by extracting gene signals using custom probe set information; HG-U133plus2_customV10.CDF (60). Resulting signal intensities were then analyzed across sample groups to calculate average fold intensity differences and significance using unpaired two-tailed *t*-test analyses with resultant ranking for FDR *q*-values using Morpheus software (Broad Institute, https://software.broadinstitute.org/morpheus). For analysis of peripheral blood cell subsets, gene expression data was directly downloaded from the Differentiation Map Portal (Broad Institute, http://www.broadinstitute.org/dmap/; also available from GEO database, GSE24759) (53).

### Tonsil and Blood Processing, Cryopreservation, and Thawing

Discarded, anonymized tonsil specimens were obtained from routine tonsillectomies performed at Children's Hospital Boston, in accordance with the Partners Institutional Review Board (IRB), which deemed the research as not meeting the definition of human subjects research. Tonsils were briefly (<1 h) stored on ice in isotonic saline solution before being transferred to Hank's Balanced Salt Solution (HBSS) for processing. Tonsils were subsequently minced in HBSS, mashed with a 5 mL syringe plunger into a 70 um nylon mesh, and removed to a conical tube stored on ice. Mononuclear cells were isolated from the interface following density gradient centrifugation through Histopaque 1077 (Sigma) at 1,000 × g in an Allegra X-12R centrifuge, without the brake. The cells were then washed 3x with cold HBSS and frozen in 90% FBS/10% DMSO freezing media in a Mr. Frosty at −80°C, before being transferred to liquid nitrogen storage. As needed, cryopreserved tonsil mononuclear cells were rapidly thawed by standard procedures. Viability was routinely >80%.

Peripheral blood mononuclear cells were isolated from de-identified leukopacks acquired from the Children's Hospital Boston Blood Donor center. Buffy coats were removed following density gradient centrifugation, washed, and frozen for later use, as described above for tonsil.

### Flow Cytometry Sorting for Gene Expression Analysis

Tonsil mononuclear cells were thawed, washed, and counted as described above. To exclude apoptotic and necrotic cells, cells were first stained with Zombie NIR fixable viability dye (Biolegend) for 15 min at room temperature in PBS. Cells were then washed and stained with a cocktail of surface stain antibodies, including anti-IgD, anti-CD27, anti-CD38, anti-CD19, anti-CD3, and anti-CD14 (all from Biolegend), and incubated for 45 min on ice. Subsequently, cells were washed two times, passed through a 35 μm nylon mesh and sorted on a BD FACS Aria II at the Harvard Division of Immunology Flow Cytometry Core. After electronically gating on lymphocytes by forward and side scatter properties and eliminating cell doublets, B cells was gated as in Figure 1A. For peripheral blood B cells, naïve B cells were gated as follows: CD19+ CD3- CD14- IgD+ CD27- cells (naïve), CD19+ CD3- CD14- IgD- CD27+ (memory). In both cases, the CD27 gate was set using a fluorescence minus one (FMO) gating control. Sorted cells were pelleted, washed 2x with PBS, then lysed for RNA extraction in Buffer RLT (Qiagen).

### Quantitative Real-Time Reverse Transcription PCR (qRT-PCR)

For gene expression analysis of tonsil or peripheral blood B cells by qRT-PCR, B cell subsets were flow cytometrically sorted to >95% purity, washed, and lysed in Buffer RLT (as described above) before RNA isolation using the RNeasy Mini (naïve, GC, memory) or Micro (plasmablast) isolation kit (Qiagen), according to the manufacturer's instructions. For cell lines, cells were isolated during log phase of growth. RNA concentration and purity were checked using a BioDrop μLITE, and 0.25 μg RNA per reaction was subsequently converted to cDNA using the SuperScript VILO cDNA synthesis kit (ThermoFisher), per the manufacturer's instructions. Samples were assayed using Fast SYBR Green Master Mix (Applied Biosystems), and kinetic PCR was performed on a StepOne Plus Real-Time PCR System (Applied Biosystems). Samples were assayed in triplicate. Data was normalized to the housekeeping gene Valosin-containing protein (*VCP)*. Relative transcript levels were analyzed using the 2^(−ΔΔ*Ct*)^ method (61). Primer sequences used can be found in Supplementary Table 1.

### Magnetic Enrichment of Naive B Cells and GC B Cells for Western Blot

Tonsil mononuclear cells were labeled for 10 min on ice with anti-CD77-FITC and anti-IgD-biotin antibody (Biolegend) in MACS buffer (PBS + 0.5% BSA + 2 mM EDTA), followed by washing and labeling in anti-biotin microbeads (Miltenyi) for 20 min on ice, per manufacturer's instructions. Cells were washed, resuspended in MACS buffer and fractionated on LS columns to collect labeled population (naïve-enriched). The unlabeled population was subsequently labeled with anti-FITC microbeads (Miltenyi) for 20 min on ice, washed, and loaded onto LS columns to isolate the GC-enriched fraction. Post-sort B cell purity was confirmed on a FACS Canto I using the flow cytometry staining procedures described above, and were at least 85% pure, but typically >90%. Naïve- and GC-enriched fractions were washed 3x in PBS before lysis in 2% NP-40 buffer / Buffer A (150 mM NaCl, 0.5 mM Tris, 1 mM EDTA) supplemented with protease/phosphatase inhibitors (Protease/Phosphatase Inhibitor Mini tablets, Thermo).

### Plant Lectin and CD45 Glycoform Staining by Flow Cytometry

Tonsil mononuclear cells were thawed, washed, and counted as described above. For cell lines, cells were grown as described above and harvested in log phase of growth. Fresh media was consistently added 1 day before the experiment to ensure adequate nutrients for proper glycosylation. Dead cells were stained by Zombie NIR fixable viability dye (Biolegend) in PBS for 15 min at room temperature (for tonsil cells only), followed by washing and staining in one of several biotinylated or FITC-conjugated plant lectins: *Arachis hypogaea* (peanut) agglutinin (PNA, Sigma), *Maackia amurensis* lectin-II (MAL-II), *Solanum tuberosum* agglutinin (STA), Jacalin lectin, *Helix pomatia* agglutinin (HPA), or *Phaselous vulgaris* leucoagglutinin (PHA-L) (all from Vector) for 45 min on ice in 1% bovine serum albumin (BSA) in PBS. For biotinylated lectins, cells were washed and subsequently incubated in Streptavidin-fluorophore conjugate for 30 min in 1% BSA in PBS on ice. Alternatively, cells were incubated in biotinylated anti-CD45 antibody (B220 (BD) or MEM55 clone (Thermo) followed by detection with Streptavidin-fluorophore conjugate, or directly assayed with FITC-conjugated MEM55 (Thermo). For analysis of total CD45 levels, APC-conjugated CD45 mAb (HI30 clone, Biolegend) was used. For tonsil cells, cells were subsequently washed and stained using a panel of surface stain lineage antibodies, including anti-IgD-FITC (or APC), anti-CD19-PerCP, anti-CD3-APC/Cy7, anti-CD14-APC/Cy7, anti-CD27-PE/Cy7, and anti-CD38-PE (all from Biolegend). For dual MEM55 and B220 or MEM55 and STA stains, cells were jointly incubated with MEM55-FITC (Thermo) and biotinylated B220 (BD) or STA (Vector), followed by detection with Streptavidin-APC conjugate, and surface stain using anti-IgD-PE, anti-CD19-APC Fire 750, anti-CD27-PE/Cy7, anti-CD38-PerCP/Cy5.5 (all from Biolegend). After staining, cells were immediately acquired on a BD FACSCanto I. Analysis was performed using FlowJo software (TreeStar). Cells were gated electronically for lymphocytes and doublet discrimination, followed by gating on B cells as shown in Figure 1A. For CD27 stains, a PE/Cy7 FMO gating control was employed. The geometric mean was used for calculation of mean fluorescence intensities (MFI) unless otherwise indicated.

### Near-Infrared Western and Lectin Blots

For sample preparation for lectin and protein immunoblots, B cell lines or magnetically-enriched primary naïve/GC B cells were washed 3x before lysis in ice-cold 2% NP-40 buffer/Buffer A (150 mM NaCl, 0.5 mM Tris, 1 mM EDTA) supplemented with protease/phosphatase inhibitors (Protease/ Phosphatase Inhibitor Mini tablets, Thermo). Debris was pelleted by centrifugation and samples were quantitated by BCA assay (Thermo) to ensure equal loading. Lysates were boiled for 10 min in Laemlli reducing sample buffer. Equivalent amounts of lysate (10–30 μg per lane) were resolved on 4–20% Criterion Tris-HCl polyacrylamide gels (BioRad), followed by transfer to 0.2 μm pore-size nitrocellulose membranes for immunoblot. Membrane blocking was performed in Odyssey Blocking buffer (Li-cor) for at least 1 h (or overnight) at room temperature. For primary antibody or lectin stain, blots were incubated in antibody / lectin stain overnight at 4°C. Staining reagents were diluted in Tris-buffered saline (pH 7.4) + Tween 20 (0.1%), diluted 1:1 in Odyssey Blocking buffer (Li-Cor). Primary reagents were detected using anti-mouse IgG (H+L), anti-rabbit IgG (H+L), or Streptavidin IR-Dye 680 or 800 CW conjugates. Blots were scanned and recorded using an Odyssey CLx Near-infrared Imaging System (Li-Cor). For dual stains, the blot was first probed and recorded with the lectin (PNA or MAL-II) in the 800 nm channel, and then subsequently re-probed and scanned with anti-CD45 antibody (Biolegend) in the 680 nm channel.

### PNA Immunoprecipitation

For PNA immunoprecipitation experiments, 30 μL of PNA-agarose beads (4.4 mg/mL PNA) were pre-blocked in 0.1% BSA, washed, and mixed with 100 μg Raji, Ramos, or SUDHL-4 lysate generated as described above. PNA-reactive glycoprotein-bound beads were immunoprecipitated overnight at 4°C, on a rotator, washed 3x with lysis buffer, then eluted by boiling in Laemmli reducing sample buffer. As a control, where indicated, immunoprecipitations were performed in the presence of 0.1 M lactose. Equal volumes of immunoprecipitated material were subsequently subjected to SDS-PAGE and Western/lectin blot with either PNA-biotin (Sigma) or mouse anti-human CD45 (Biolegend), followed by fluorophore conjugated secondary reagents (Li-Cor).

### Enzymatic Removal of Cell Surface Sialic Acids

Cleavage of cell surface sialic acids was performed on live tonsil mononuclear cells or B cell lines using *A. ureafaciens* sialidase (Roche, [final] = 125 mU mL^−1^) in serum-free RPMI for 1 h at room temperature. Cells were pelleted and washed 2x before proceeding with flow cytometric staining. Effective removal of sialic acid removal was confirmed by flow cytometric staining with *Sambucus nigra agglutinin* and *Maackia amurensis agglutinin*-II.

### Cellular O-glycome Reporter Analysis

Cells and glycans were prepared for Cellular O-glycome Reporter Analysis as previously described (46). Briefly, Ramos untransduced, vector control-transduced, or ST3Gal1OE-transduced B cells were seeded at 0.3 × 10^6^ mL^−1^ in tissue culture medium (with 5% FBS) in six well tissue culture plates. Peracetylated Benzyl α-D-GalNAc (Ac3GalNAc-α-Bn) was added to each well to a final concentration of 50 μM. Cells were grown for 72 h, followed by cell pelleting and collection of media.

To purify glycans from media, media was filtered through a 10-kDa centrifugal filter (Amicon Ultra 4, Millipore) for 30 min at 2,465 × g. Bn-containing O-glycans were purified using a Sep-Pak-3-cc C18 cartridge (Waters). To equilibrate the column, 2 mL acetonitrile was applied two times followed by four washes with 2 mL 0.1% (v/v) trifluoroacetic acid (TFA). Glycan-containing media was then added to the column, followed by four washes with 2 mL 0.1% (v/v) TFA. To elute Bn-containing O-glycans, 1.5 mL 50% (v/v) acetonitrile, 0.1% (v/v) TFA was applied to the column two times. Organic solvents were evaporated by SpeedVac, and the samples were lyophilized prior to MS analysis.

### Preparation of Cells for N- and O-glycomic Analysis

Ramos untransduced, vector control-transduced, and ST3Gal1OE-transduced B cells were harvested in the log phase of growth (0.75–1.25 × 10^6^ cells mL^−1^), pelleted, washed in excess PBS two times, and media completely aspirated. Cell pellets (20 × 10^6^ cells per condition) were snap frozen in a dry ice / isopropanol slurry for 5 min and immediately stored at −80°C prior to MS analysis.

### Glycomics Analysis of Ramos B Cells

For N- and conventional O-glycan structural analysis of untransduced, control and ST3Gal1OE Ramos B cells were treated as described previously (48, 62). Briefly, cell pellets were subjected to sonication in the presence of detergent (CHAPS), reduced in 4 M guanidine-HCl (Pierce), carboxymethylated, and digested with porcine trypsin (Sigma). The digested glycoproteins were then purified by C_18_-Sep-Pak (Waters Corp., Hertfordshire, UK). N-glycans were released by peptide N-glycosidase F (E.C. 3.5.1.52; Roche Applied Science) digestion, whereas O-glycans were released by reductive elimination. Released N- and O-glycans were permethylated using the sodium hydroxide procedure and purified by C_18_-Sep-Pak. Purified permethylated N- and O-glycans were found on the 50% acetonitrile fraction. The results shown are representative of two independent cell glycan preparations.

For CORA O-glycan structural analysis of untransduced, empty vector control and ST3Gal1OE Ramos B cells were treated as described previously (46). Isolated Bn-O-glycans were permethylated using the sodium hydroxide procedure and purified by C_18_-Sep-Pak as described above for the conventional O-glycan structural analysis. Purified permethylated Bn-O-glycans were found on the 50% acetonitrile fraction. The results shown are representative of two independent cell glycan preparations.

Matrix-assisted laser desorption ionization-time of flight mass spectrometry (MALDI-TOF MS) and MALDI-TOF/TOF MS/MS were employed to analyze the structure of all above permethylated released glycans. MS and MS/MS data were acquired using a 4800 MALDI-TOF/TOF (Applied Biosystems Sciex) mass spectrometer. Permethylated samples were dissolved in 10 μl of methanol, and 1 μl of dissolved sample was premixed with 1 μl of matrix (10 mg/ml 3,4-diaminobenzophenone in 75% (v/v) aqueous acetonitrile), spotted onto a target plate, and dried under vacuum. For the MS/MS studies, the collision energy was set to 1 kV, and argon was used as collision gas. The 4,700 Calibration standard kit, calmix (Applied Biosystems Sciex), was used as the external calibrant for the MS mode, and [Glu1] fibrinopeptide B human (Sigma) was used as an external calibrant for the MS/MS mode. The MS and MS/MS data were processed using Data Explorer 4.9 Software (Applied Biosystems). The processed spectra were subjected to manual assignment and annotation with the aid of a glycobioinformatics tool, GlycoWorkBench (63). The proposed assignments for the selected peaks were based on ^12^C isotopic composition together with knowledge of the biosynthetic pathways. The proposed structures were then confirmed by data obtained from MS/MS and linkage analysis experiments.

### Statistical Analysis

Statistical analyses were performed using Prism 7.0 software (GraphPad). For tests involving two groups, hypothesis testing was carried out using Welch's unpaired two-tailed *t*-test. For hypothesis testing of groups of three or more samples, and when variance was found to be not significantly different by *F*-test, a one-way analysis of variance (ANOVA) test was used with Tukey's correction for multiple comparisons. Where variances were unequal, a Kruskal–Wallis test was used instead with Dunn's correction for multiple comparisons. Bars and errors bars always depict the mean or standard error of the mean (SEM) from biological replicates, respectively, unless otherwise indicated. *P*-values < 0.05 were considered statistically significant.

## Data Availability Statement

The following datasets analyzed in this study are available on the Gene Expression Omnibus website (https://www.ncbi.nlm.nih.gov/geo/) under the following identifiers: GSE12195 (tonsil B cell expression analysis) (30); GSE24759 (hematopoietic cell expression analysis) (53). Hematopoietic cell expression data is also accessible at the following link: http://www.broadinstitute.org/dmap/home.

## Ethics Statement

This study was carried out in accordance with the recommendations of the Partners Institutional Review Board, which deemed the work as not meeting the definition of human subjects research.

## Author Contributions

NG and CD conceived the study. NG and AA performed the experiments and analyzed the data. NG, SB, and HW generated the ST3Gal1 cell lines. MK and RC provided technical assistance and expertise with CORA O-glycomics analysis. AA and SH performed O-glycomic analyses. GL assisted with tonsil tissue acquisition. NG, AA, JL, JG, SK, AD, SB, HW, SH, and CD contributed intellectually to the study. SH and AD supervised MS glycomics assessments. CD supervised the entire study. NG, AA, SH, and CD wrote the manuscript.

### Conflict of Interest Statement

The authors declare that the research was conducted in the absence of any commercial or financial relationships that could be construed as a potential conflict of interest.

## Abbreviations

GC: germinal center
PNA: peanut agglutinin
MAL-II: *Maackia amurensis* lectin II
SNA: *Sambucus nigra* agglutinin
STA: *Solanum tuberosum* agglutinin
HPA: *Helix pomatia* agglutinin
PHA-L: *Phaseolus vulgaris* leuco-agglutinin
OE: overexpressing
DLBCL: diffuse large B cell lymphoma
IP: immunoprecipitation
MS: mass spectrometry
CORA: Cellular O-glycome Reporter Amplification
Gal: galactose
GalNAc: N-acetylgalactosamine
poly-LacNAc: poly-N-acetyllactosamine
mAb: monoclonal antibody.
