# Supplementary material for: Human B Cell Differentiation Is Characterized by Progressive Remodeling of O-Linked Glycans
Source: Front Immunol. 2018 Dec 14;9:2857. doi: 10.3389/fimmu.2018.02857 (PMC6302748; doi:10.3389/fimmu.2018.02857)
Supplement: Supplementary file 2 [file Image_1.pdf]

Supplementary Material

Human B cell differentiation is characterized by progressive remodeling of O-linked glycans

Nicholas Giovannone, Aristotelis Antonopoulos, Jennifer Liang, Jenna Geddes Sweeney, Matthew R. Kudelka, Sandra L. King, Gi Soo Lee, Richard D. Cummings, Anne Dell, Steven R. Barthel, Hans R. Widlund, Stuart M. Haslam, and Charles J. Dimitroff*

*** Correspondence:** Charles J. Dimitroff, cdimitroff@bwh.harvard.edu

1.1 Supplementary Figures

Supplementary Figure 1

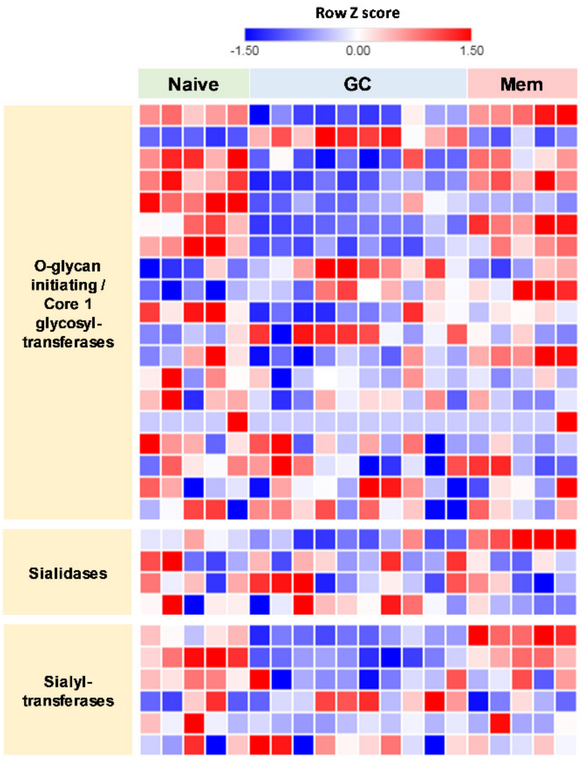

Gene	N vs. GC T score	N vs. GC *p*-value	N vs. GC FDR (BH)	GC vs M T score	GC vs M *p*-value	GC vs M FDR (BH)
GALNT1	8.66	0.00	0.00 *	-6.82	0.00	0.00 *
GALNT14	-10.01	0.00	0.00 *	7.05	0.00	0.00 *
C1GALT1	5.69	0.00	0.00 *	-3.95	0.00	0.01 *
GALNT11	7.52	0.00	0.00 *	-6.89	0.00	0.00 *
GALNT6	5.79	0.00	0.01 *	0.04	0.97	0.97
GALNT10	5.50	0.00	0.01 *	-7.51	0.00	0.00 *
GALNT3	4.68	0.01	0.02 *	-5.45	0.00	0.00 *
C1GALT1C1	-3.67	0.01	0.02 *	12.45	0.04	0.09
GALNT12	-3.48	0.01	0.02 *	-2.04	0.09	0.17
GALNT2	3.03	0.02	0.05	-1.88	0.09	0.17
GALNTL4	-1.79	0.10	0.21	1.44	0.17	0.30
GALNT7	1.84	0.12	0.23	-5.47	0.00	0.00 *
GALNTL1	1.31	0.25	0.39	0.08	0.93	0.97
GALNT9	1.14	0.31	0.45	0.20	0.85	0.97
GALNT5	1.00	0.37	0.49	-1.00	0.37	0.59
GALNT8	0.94	0.37	0.49	0.64	0.53	0.78
GALNTL2	0.62	0.55	0.66	-0.22	0.83	0.97
GALNTL5	-0.35	0.73	0.84	-0.03	0.97	0.97
GALNT13	0.19	0.86	0.89	-0.49	0.64	0.86
NEU1	3.02	0.01	0.02 *	-7.47	0.00	0.00 *
NEU2	0.34	0.75	0.84	1.10	0.29	0.29
NEU3	-0.23	0.82	0.88	1.16	0.28	0.29
NEU4	-0.04	0.97	0.97	1.71	0.11	0.22
ST3GAL5	5.32	0.00	0.00 *	-7.58	0.00	0.00 *
ST3GAL1	6.26	0.00	0.01 *	-7.82	0.00	0.00 *
ST3GAL3	1.59	0.14	0.25	-0.46	0.66	0.73
ST3GAL2	-1.40	0.20	0.34	2.81	0.02	0.03 *
ST3GAL6	1.36	0.25	0.39	-0.88	0.43	0.64
ST3GAL4	-0.71	0.50	0.64	0.35	0.73	0.73

Supplementary Fig. 1: Analysis of O-glycosylation enzyme expression in tonsillar B cells. Publicly available datasets (GSE12195) were analyzed for expression of O-glycan initiating enzymes (*GALNTs*), Core 1 synthase (*C1GALT1*), C1GalT1 chaperone (*C1GALT1C1*), sialidases (*NEUs*), and α2,3-sialyltransferases (*ST3GALs*) in human B cell subsets. Each column represents a unique tonsil specimen. Statistics were performed for each row by individual two-tailed, unpaired Student's *t*-test and corrected for multiple comparisons using Benjamini-Hochberg False Discovery procedure. FDR q< 0.05 (*) was considered statistically significant.

Supplementary Fig. 2

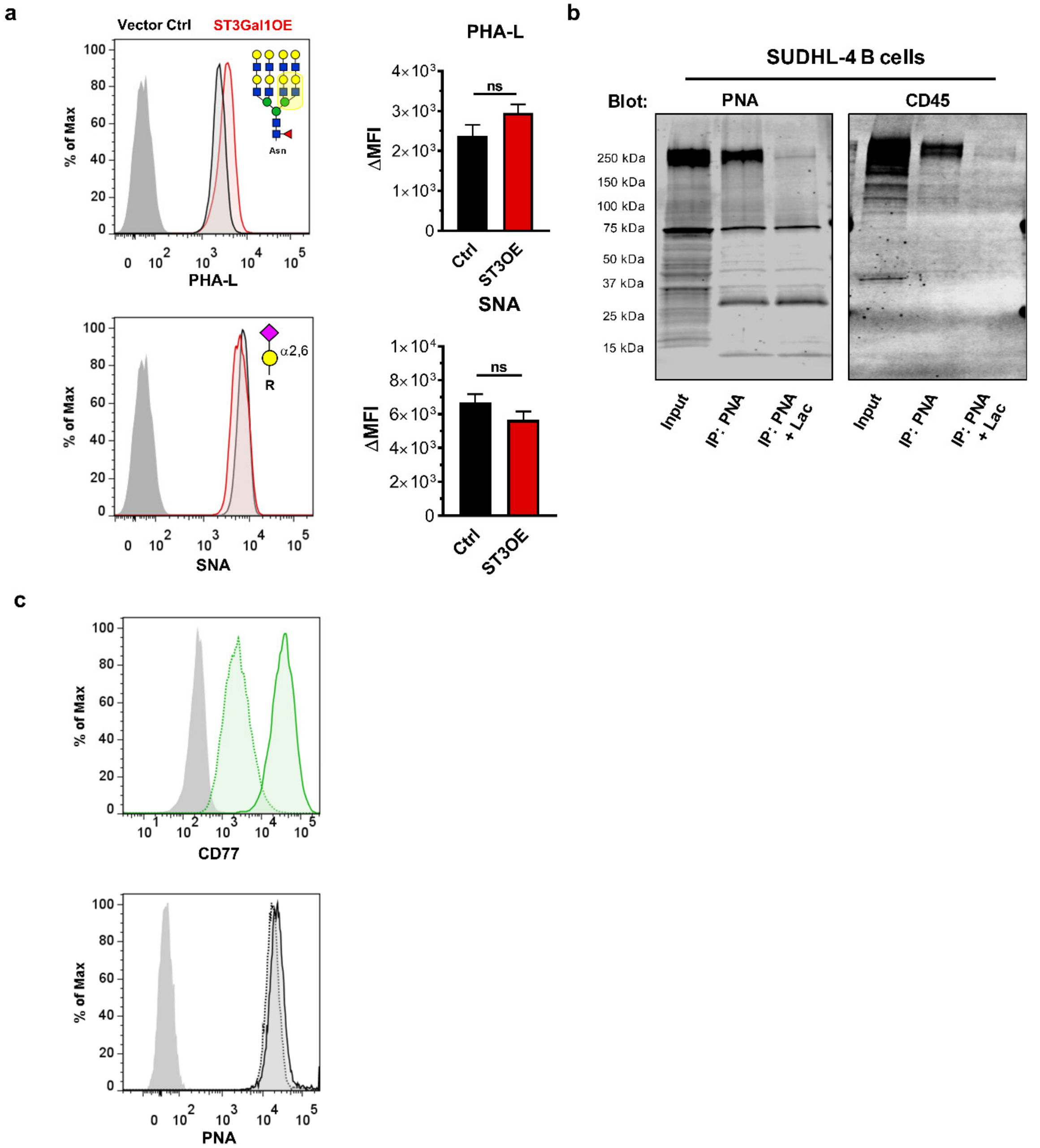

Supplementary Fig. 2: PNA ligands are expressed as O-glycans on CD45 and are not meaningfully expressed on glycolipids of Ramos B cells. (a) Representative histogram (*left*) and quantification (*right*) of PHA-L (tri- and tetra-antennary N-glycans) and SNA (α2,6-sialic acid) plant lectin binding to vector control and ST3Gal1OE Ramos B cells. **(b)** Immunoprecipitation (IP) of PNA-binding proteins from lysates of the GC-derived diffuse large B cell lymphoma (DLBCL) lymphoma cell line SUDHL-4, followed by SDS-PAGE and immunoblot with either PNA (*left*) or total CD45 antibody (*right*). As a negative control for carbohydrate binding, IP was also performed in the presence of a sugar inhibitor, lactose (Lac; right lane). **(c)** Representative histograms depicting CD77 (Gb3 glycolipid) expression and PNA binding by Ramos B cells without (solid line) or with (dotted line) 72hr treatment with D,l-threo-1-phenyl-2-hexadecanoylamino-3-pyrrolidino-1-propanol-HCl (PPPP), a Glc-Cer synthase (UCGC) inhibitor that blocks glycolipid synthesis.

Supplementary Fig. 3

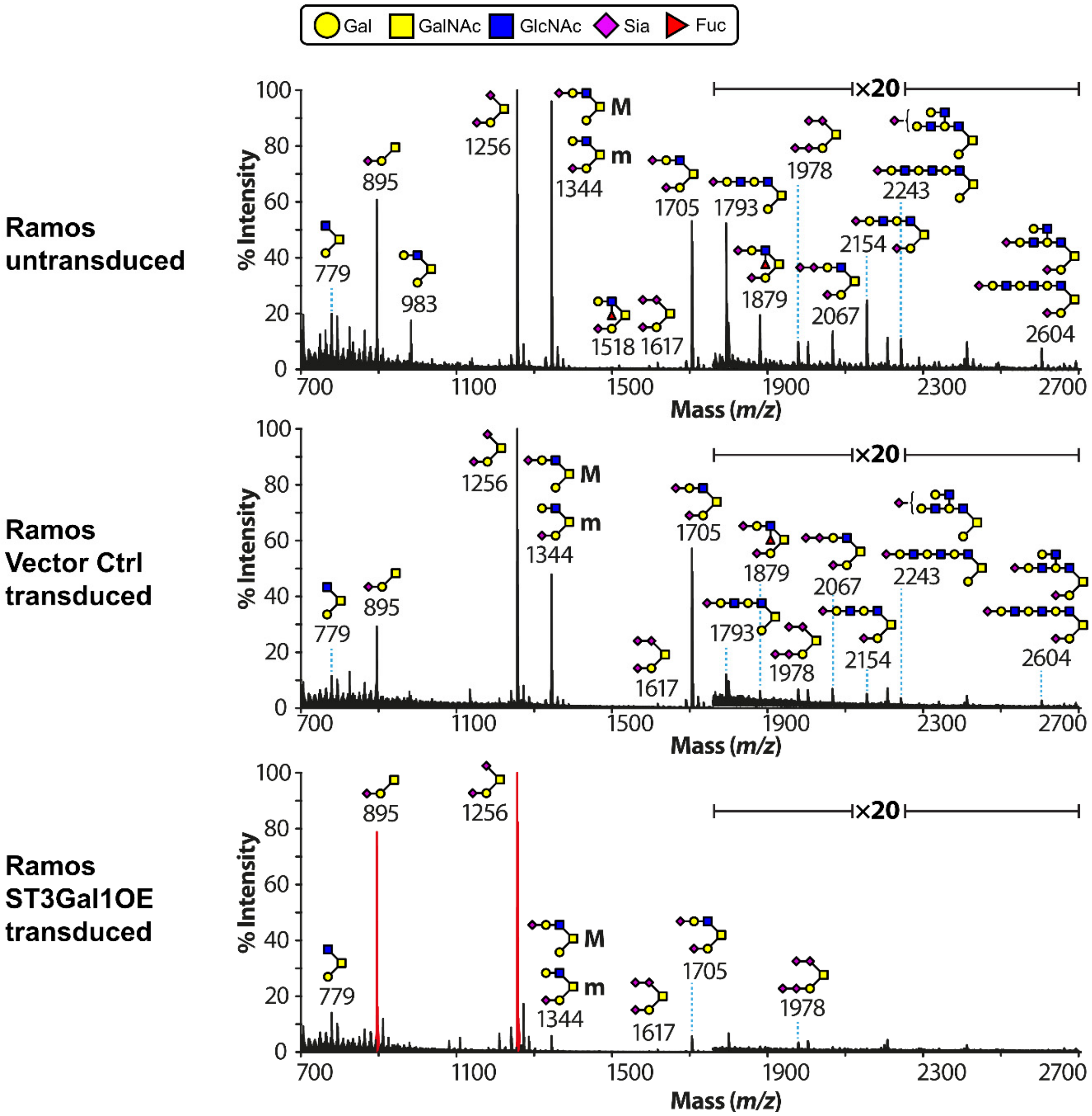

Supplementary Figure 3: O-glycomic analysis of ST3Gal1 variant Ramos B cells. Conventional MALDI-TOF MS analysis of O-glycans from untreated, vector control and ST3Gal1OE Ramos B cells. Structures above a bracket have not been unequivocally defined. Indicated areas in the spectra have a 20-fold magnification. "M" and "m" designations indicate major and minor abundances, respectively. Cartoon structures were drawn according to http://www.functionalglycomics.org guidelines and are representative from repeat experiments on two different biological replicates. Structure assignments are based on composition, tandem mass spectrometry and biosynthetic knowledge. Fuc, fucose; Man, mannose; Gal, galactose; GlcNAc, N-acetylglucosamine; GalNAc, N-acetylgalactosamine; Sia, N-acetylneuraminic acid (sialic acid). Full methods can be found in Materials and Methods.

Supplementary Fig. 4

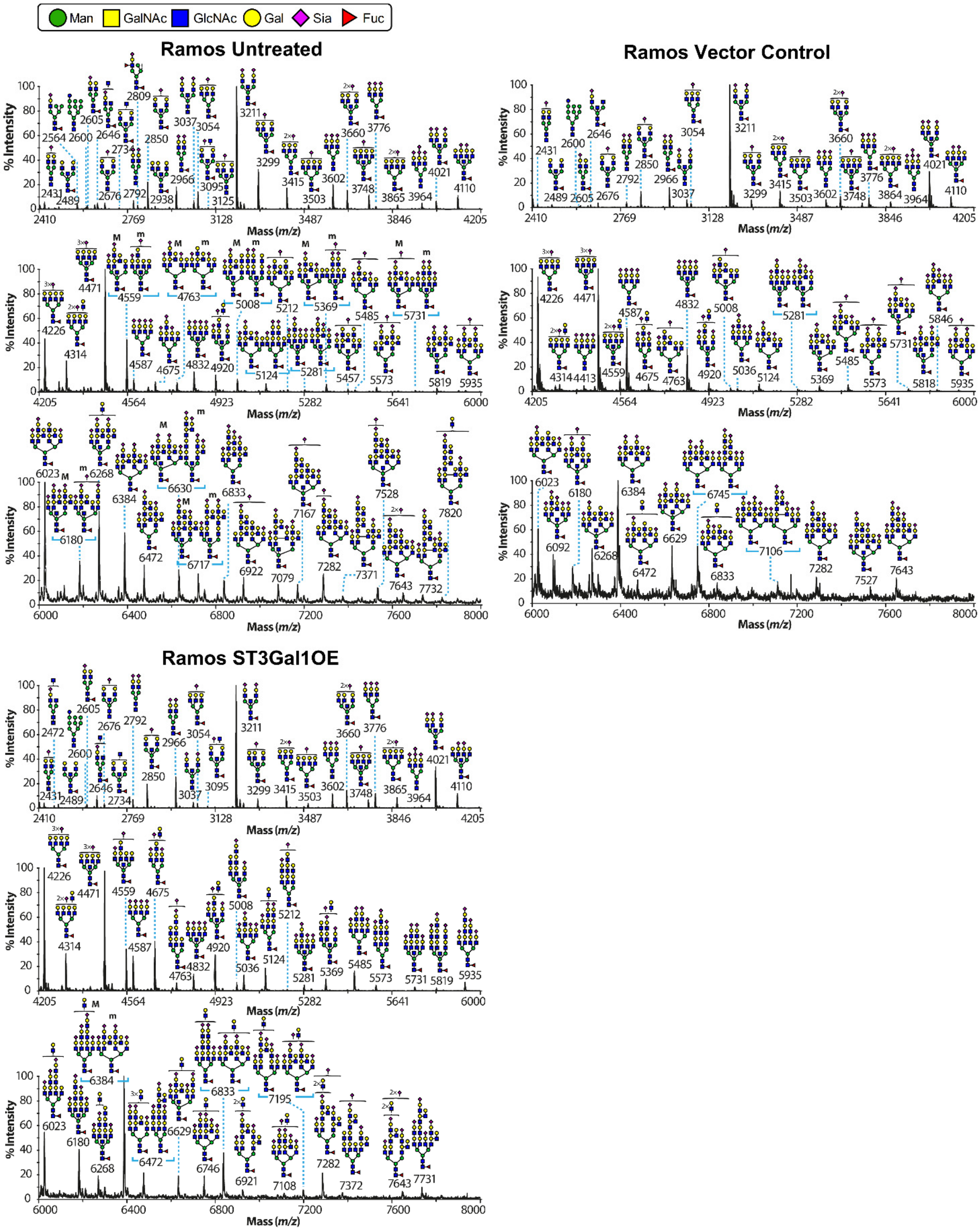

Supplementary Fig. 4: N-glycomic analysis of ST3Gal1 variant Ramos B cells. MALDI-TOF MS analysis of permethylated N-glycans released by PNGase F digestion of untreated, vector control and ST3Gal1OE Ramos B cells. Structures above a bracket have not been unequivocally defined. “M” and “m” designations indicate major and minor abundances, respectively. Cartoon structures were drawn according to http://www.functionalglycomics.org guidelines and are representative from repeat experiments on two different biological replicates. Structure assignments are based on composition, tandem mass spectrometry and biosynthetic knowledge. Fuc, fucose; Man, mannose; Gal, galactose; GlcNAc, N-acetylglucosamine; Sia, N-acetylneuraminic acid (sialic acid). Full methods can be found in Materials and Methods.

Supplementary Fig. 5

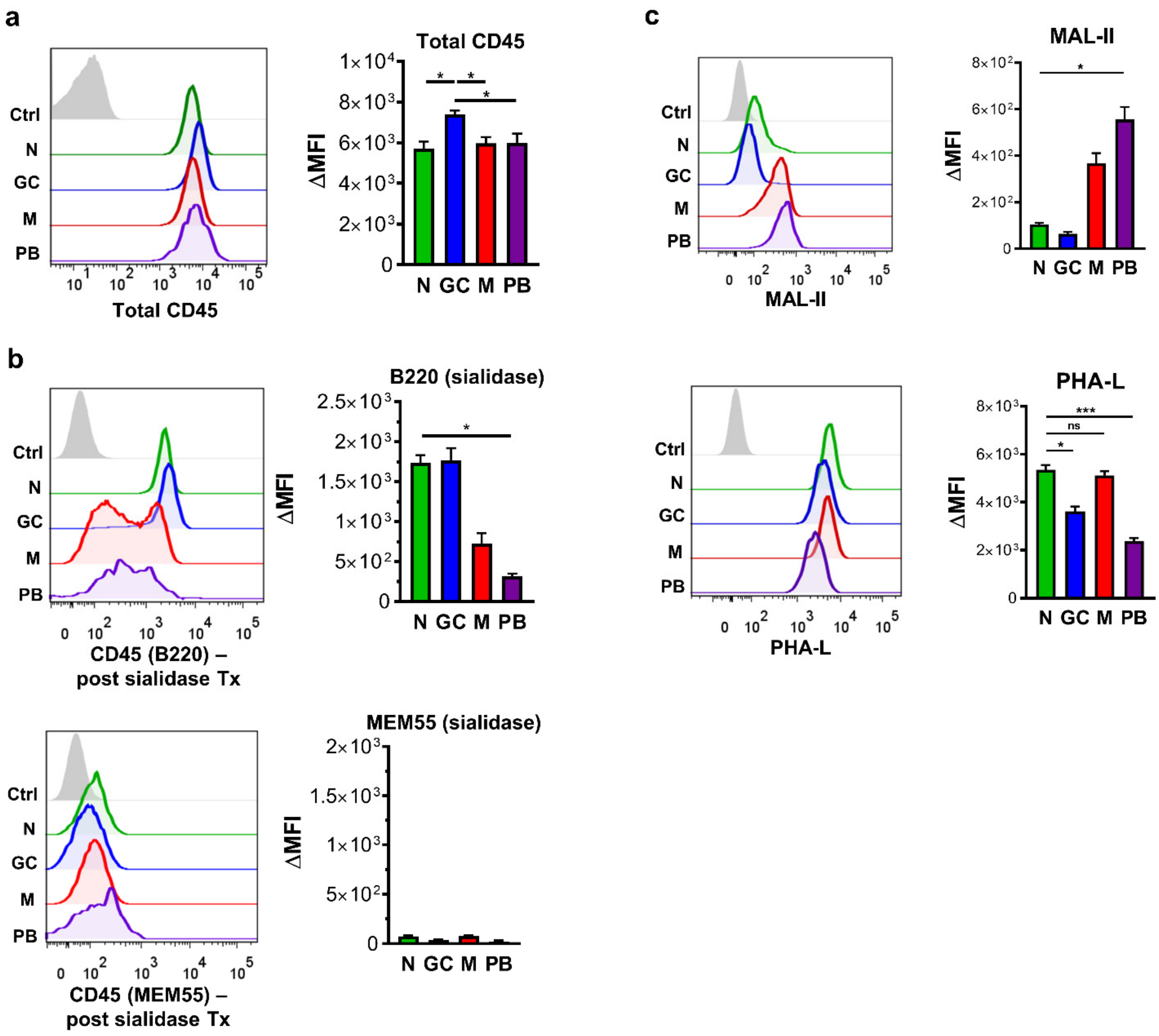

Supplementary Fig. 5: Analysis of CD45 glycoform and global glycosylation of primary B cells by CD45 mAb and plant-lectin based flow cytometry. (a) Representative histograms (*left*) and quantification (*right*) of total CD45 expression (HI30 clone) on tonsillar B cell subsets. **(b)** Representative histograms (*left*) and quantification (*right*) of binding of CD45 mAbs B220 and MEM55 to the indicated tonsillar B cell subsets following treatment with *Arthrobacter ureafaciens* sialidase. **(c)** Representative histograms (*left*) and quantification (*right*) of binding of MAL-II (sialylated T-antigen) and PHA-L plant lectins (tri- and tetra-antennary complex N-glycan binding preference) to primary tonsillar B cells by flow cytometry, gated as in Fig.1a. See also Fig. 7a for schematic depicting glycan-binding preferences of MAL-II and PHA-L. For (a) and (b), n=5 distinct tonsil specimens. For (c), n=8 (MAL-II) or n=9 (PHA-L) distinct tonsil specimens. Statistics in (a-c) were calculated using a Kruskal-Wallis test with Dunn's multiple comparisons test. Throughout, bars and error bars depict the mean and SEM, respectively. ns = not significant, *$p \leq 0.05$, ***$p \leq 0.001$. ΔMFI, background subtracted geometric mean fluorescence intensity.

Supplementary Table 1: Oligonucleotide sequences

Target	Application	Forward (5'→3')	Reverse (5'→3')
Hu *ST3GAL1*	cDNA Amplification	cgacgaattcgccacc**atggtgaccctgcggaagagg**	ccgggatcc**tcatctccccttgaagatccggatttt**
Hu *ST3GAL1*	qRT-PCR (60ºC)	gcatttctctttcccacagc	ctaattcccagccaccttca
Hu *GCNT1*	qRT-PCR (60ºC)	aatttccgatgcccatgat	agggccaagtcctttcaaat
Hu *VCP* (housekeeping)	qRT-PCR (60ºC)	aggatgatccagtgcctgag	ggaatctgaagctgccaaag

Supplementary Table 2: Antibodies and reagents

**Lectins and glycobiology reagents**						
**Target**	**Conjugate**	**Clone**	**Source**	**Catalog number**	**Concentration / Dilution**	**Incubation time**
Arachis hypogea (PNA)	FITC	-	Sigma	L7381	10μg/mL (FACS)	45min, ice
Jacalin lectin	FITC	-	Vector	FL-1151	5μg/mL (FACS)	45min, ice
Phaseolus vulgaris Leucoagglutinin (PHA-L)	FITC	-	Vector	FL-1111	2μg/mL (FACS)	45min, ice
Solanum Tuberosum Agglutinin (STA)	FITC	-	Vector	FL-1161	2μg/mL (FACS)	45min, ice
Helix pomatia agglutinin	AlexaFluor 488	-	Life Technologies	L11271	5μg/mL (FACS)	45min, ice
Arachis hypogea (PNA)	Biotin	-	Vector	L6135	2μg/mL (FACS)	45min, ice
Sambucus Nigra Agglutinin (SNA)	Biotin	-	Vector	FL-1301	2μg/mL (FACS)	45min, ice
Maackia Amurensis Lectin II (MAL-II)	Biotin	-	Vector	B-1265	0.5μg/mL (FACS)	45min, ice
Phaseolus vulgaris Leucoagglutinin (PHA-L)	Biotin	-	Vector	B-1115	0.1μg/mL (FACS)	45min, ice
Solanum Tuberosum Agglutinin (STA)	Biotin	-	Vector	B-1165	0.1μg/mL (FACS)	45min, ice
Sambucus Nigra Agglutinin (SNA)	Biotin	-	Vector	B-1305	0.25μg/mL (FACS)	45min, ice
Arthrobacter ureafaciens sialidase	-	-	Millipore-Sigma	10269611001	125mU / mL	1hr, RT
D-1-threo-1-phenyl-2-hexadecanoylamino-3-pyrrolidino-1-propanol-HCl (PPPP)	-	-	Gift from Dr. Ronald L. Schnaar (Johns Hopkins)	-	2μM	72hr incubation

Flow cytometry antibodies and staining reagents						
**Target**	**Conjugate**	**Clone**	**Source**	**Catalog number**	**Concentration / Dilution**	**Incubation time**
CD3	APC-Cy7	HIT3a	Biolegend	300318	1:100 (FACS)	45min, ice
CD14	APC-Cy7	HCD14	BIolegend	325620	1:160 (FACS)	45min, ice
CD19	PerCP	HIB19	Biolegend	302228	1:40 (FACS)	45min, ice
CD19	APC	HIB19	Biolegend	302212	1:100 (FACS)	45min, ice
CD19	APC/Fire 750	HIB19	Biolegend	302257	1:40 (FACS)	45min, ice
CD27	PE-Cy7	LG.3A10	Biolegend	124216	1:160 (FACS)	45min, ice
CD38	PE	HB-7	Biolegend	356604	1:160 (FACS)	45min, ice
CD38	PerCP/Cy5.5	HB-7	Biolegend	356613	1:160 (FACS)	45min, ice
CD43 (Core 2 glycoform)	-	1D4	LSBio	LSC179306	1:500 (FACS)	45min, ice
CD45	APC	HI30	Biolegend	304012	1:25 (FACS)	45min, ice
CD45 (B220)	Biotin	RA3-6B2	BD	553086	1:100 (FACS)	45min, ice
CD45RB (MEM55)	-	MEM55	Thermo	MA1-19115	1:500 (FACS)	1hr, RT
CD45RB (MEM55)	FITC	MEM55	Thermo	MA1-19571	1:5 (FACS)	45min, ice
IgD	FITC	IA6-2	Biolegend	348206	1:200 (FACS)	45min, ice
IgD	PE	IA6-2	Biolegend	348203	1:200 (FACS)	45min, ice
Streptavidin	FITC	-	Biolegend	405202	1:1000 (FACS)	30min, ice
Streptavidin	APC	-	Biolegend	405207	1:500 (FACS)	30min, ice
Zombie NIR Fixable Viability Kit	-	-	Biolegend	423106	1:1600 (FACS)	45min, ice
**Magnetic sorting antibodies and reagents**						
**Target**	**Conjugate**	**Clone**	**Source**	**Catalog number**	**Concentration / Dilution**	**Incubation time**
Anti-Biotin microbeads	-	-	Miltenyi	130-090-485	Manufact. guidelines	Manufact. guidelines
Anti-FITC microbeads	-	-	Miltenyi	130-048-701	Manufact. guidelines	Manufact. guidelines
IgD	Biotin	IA6-2	Biolegend	348212	1:40 (MACS)	10min, ice
CD77	FITC	5B5	Biolegend	357104	1:20 (MACS)	10min, ice
**Western blot and immunoprecipitation reagents**						
**Target**	**Conjugate**	**Clone**	**Source**	**Catalog number**	**Concentration / Dilution**	**Incubation time**
CD45	-	HI30	Biolegend	304002	1μg/mL (WB)	1hr, RT
CD45	-	D9M8I	CST	13917	1:2000	O/N, 4C
CD45RB (MEM55)	-	MEM55	Thermo	MA1-19115	2μg/mL (WB)	1hr, RT
Arachis hypogea (PNA)	Biotin	-	Sigma	L6135	5μg/mL	1hr, RT
Maackia Amurensis Lectin II (MAL-II)	Biotin	-	Vector	B-1265	0.5μg/mL	1hr, RT
Donkey anti-Goat IgG (H+L)	IRDye® 800CW	Polyclonal	Li-Cor	926-32214	1:20,000 (WB)	30min, RT

Goat anti-Rabbit IgG (H+L)	IRDye® 800CW	Polyclonal	Li-Cor	926-32211	1:20,000 (WB)	30min, RT
Goat anti-Mouse IgG (H+L)	IRDye® 800CW	Polyclonal	Li-Cor	926-32210	1:20,000 (WB)	30min, RT
Goat anti-Rabbit IgG (H+L)	IRDye® 680LT	Polyclonal	Li-Cor	926-68023	1:20,000 (WB)	30min, RT
Goat anti-Mouse IgG (H+L)	IRDye® 680RD	Polyclonal	Li-Cor	926-68070	1:20,000 (WB)	30min, RT
Streptavidin	IRDye® 800CW	-	Li-Cor	926-32230	1:10,000 (WB)	30min, RT
